# Universal risk phenotype of US counties for flu-like transmission to improve county-specific COVID-19 incidence forecasts

**DOI:** 10.1371/journal.pcbi.1009363

**Published:** 2021-10-14

**Authors:** Yi Huang, Ishanu Chattopadhyay

**Affiliations:** 1 Department of Medicine, University of Chicago, Chicago, Illinois, United States of America; 2 Committee on Genetics, Genomics & Systems Biology, University of Chicago, Chicago, Illinois, United States of America; 3 Committee on Quantitative Methods in Social, Behavioral, and Health Sciences, University of Chicago, Chicago, Illinois, United States of America; 4 Center of Health Statistics, University of Chicago, Chicago, Illinois, United States of America; Institute for Disease Modeling, UNITED STATES

## Abstract

The spread of a communicable disease is a complex spatio-temporal process shaped by the specific transmission mechanism, and diverse factors including the behavior, socio-economic and demographic properties of the host population. While the key factors shaping transmission of influenza and COVID-19 are beginning to be broadly understood, making precise forecasts on case count and mortality is still difficult. In this study we introduce the concept of a universal geospatial risk phenotype of individual US counties facilitating flu-like transmission mechanisms. We call this the Universal Influenza-like Transmission (UnIT) score, which is computed as an information-theoretic divergence of the local incidence time series from an high-risk process of epidemic initiation, inferred from almost a decade of flu season incidence data gleaned from the diagnostic history of nearly a third of the US population. Despite being computed from the past seasonal flu incidence records, the UnIT score emerges as the dominant factor explaining incidence trends for the COVID-19 pandemic over putative demographic and socio-economic factors. The predictive ability of the UnIT score is further demonstrated via county-specific weekly case count forecasts which consistently outperform the state of the art models throughout the time-line of the COVID-19 pandemic. This study demonstrates that knowledge of past epidemics may be used to chart the course of future ones, if transmission mechanisms are broadly similar, despite distinct disease processes and causative pathogens.

## Author summary

Accurate case count forecasts in an epidemic is non-trivial, with the spread of infectious diseases being modulated by diverse hard-to-model factors. This study introduces the concept of a universal risk phenotype for US counties that predictably increases the risk of person-to-person transmission of influenza-like illnesses; universal in the sense that it is pathogen-agnostic provided the transmission mechanism is similar to that of seasonal influenza. We call this the Universal Influenza-like Transmission (UnIT) score, which accounts for unmodeled effects by automatically leveraging subtle geospatial patterns underlying the flu epidemics of the past. It is a phenotype of the counties themselves, as it characterizes how the transmission process is differentially impacted in different geospatial contexts. Grounded in information-theory and machine learning, the UnIT score reduces the need to manually identify every factor that impacts the case counts. Applying to the COVID-19 pandemic, we show that incidence patterns from a past epidemic caused by an appropriately-chosen distinct pathogen can substantially inform future projections. Our forecasts consistently outperform the state of the art models throughout the time-line of the COVID-19 pandemic, and thus is an important step to inform policy decisions in current and future pandemics.

## Introduction

We are in the midst of a global pandemic caused by the novel coronavirus SARS-CoV-2, and reliable prediction of the future local and national case count is crucial for crafting effective intervention policies. Thus the need for tools that chart the likely course of an epidemic in the human population is now felt more than ever. The spread of a transmissible virus is shaped by diverse interacting factors that are hard-to-model and respond to [1], including the specific transmission mechanism, the survivability of the pathogen outside the host under harsh environmental conditions, and the ease of access to susceptible hosts—determined in part by the density of the local population, its travel habits [1], and compliance to common-sense social distancing policies. Additionally, the prevalence of pre-existing medical conditions in the local population, and its demographic makeup, might modulate susceptibility of specific hosts to the virus, slowing or accelerating the spread of the disease [2, 3]. While a broad set of putative factors shaping the spread of communicable viruses such as the seasonal Influenza and COVID-19 are increasingly becoming clear [4–15], making precise granular actionable forecasts of the case counts over time is still difficult. At present, faced with the challenge of forecasting COVID-19 incidence over time, a diversity of modeling approaches have emerged [16–22]. However a single best model is yet to coalesce.

### Key insight

In this study we introduce the concept of a universal geospatial risk of person-to-person transmission of influenza-like illnesses in the US; universal in the sense that it is pathogen-agnostic provided the transmission mechanism is broadly similar to that of seasonal Influenza. We call this the Universal Influenza-like Transmission (UnIT) score. Transmission dynamics in the general population is known to be modulated by diverse factors, only a few of which have been investigated, and are now beginning to be characterized. In all likelihood many unmodeled factors remain, along with the impact of non-trivial interactions between such known and unknown covariates that are hard to disentangle and account for. The UnIT score allows us to account for the impact of these unmodeled effects by automatically leveraging subtle emergent geospatial patterns underlying the seasonal flu epidemics of the past. In particular, we reduce the need for human modelers to manually identify every putative covariate that impacts the process.

Importantly, the UnIT score—once computed—has applicability beyond seasonal influenza. Validating our claim that the estimated UnIT score indeed quantifies a risk phenotype of individual counties for a disease with a flu-like transmission mechanism, we significantly improve incidence forecasts for COVID-19 over currently proposed state of the art models. We show that the UnIT score emerges as the most important factor “explaining” observed county-specific incidence trends for COVID-19 in the US, with coefficients in normalized multi-variate regression dominating those for typical covariates. Thus, our key insight is that incidence patterns from a past epidemic caused by a different pathogen can substantially inform current projections under mild assumptions on the similarity of the transmission mechanisms. We operationalize this insight by crafting a general information-theoretic principle to transfer this past knowledge to the new context of COVID-19. This is accomplished via a new computable measure of intrinsic similarity between stochastic sample paths generated by the hidden processes driving incidence.

### Modeling approach

**Fig 1:**
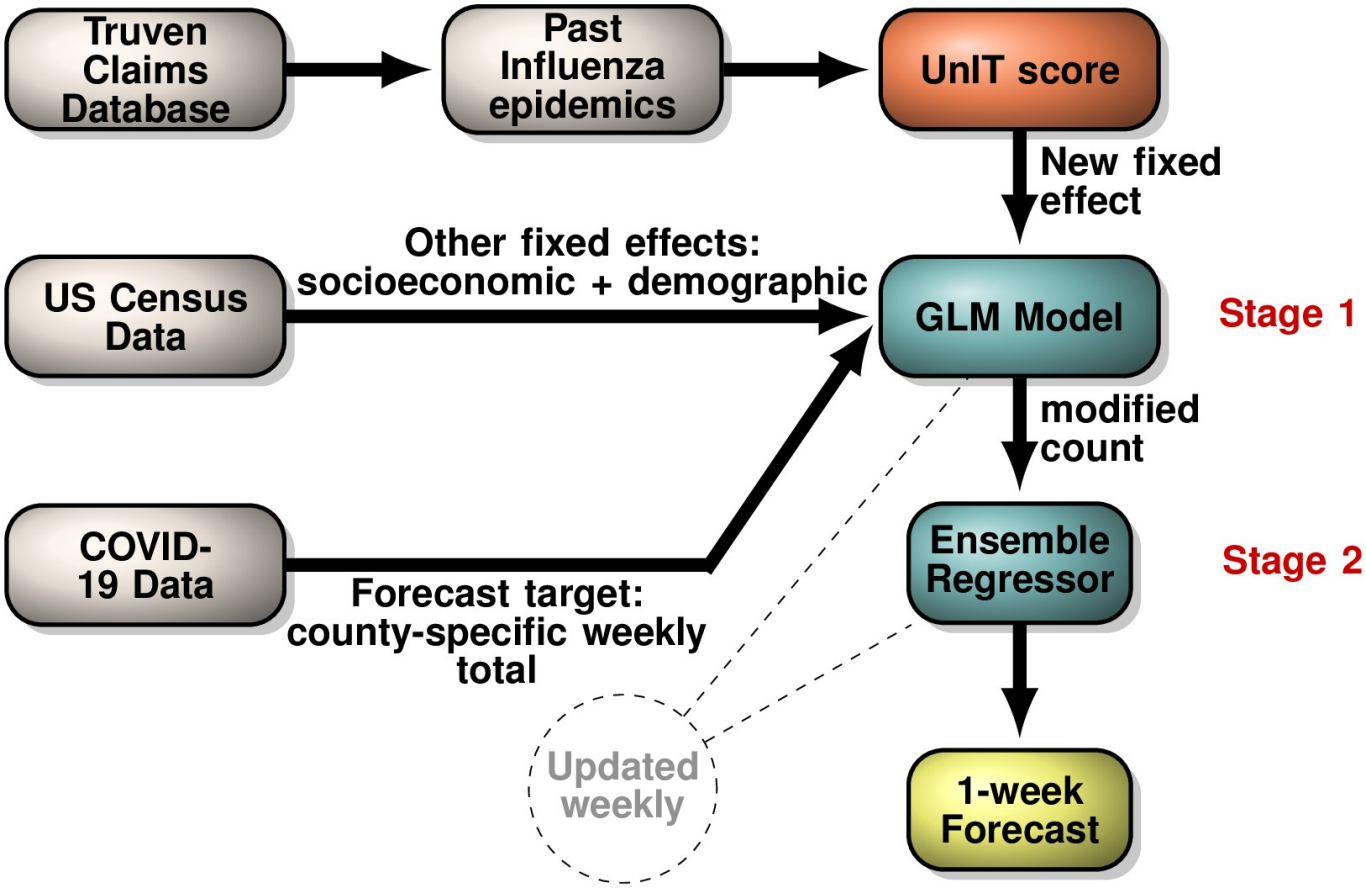
Modeling scheme. We use a national insurance claims database with more than 150 million people tracked over a decade (Truven Claims database) to curate geospatial incidence records for past Influenza epidemics over nearly a decade, which informs our new UnIT score. This score is then used as an additional fixed effect along with other putative socio-economic and demographic covariates obtained from US Census to infer a General Linear Model (GLM) explaining the weekly county-specific case COVID-19 case count. Using this inferred GLM model we “correct” the observed weekly case count, and use it as the only feature in an ensemble regressor to forecast county-specific count totals. The GLM model and the regressor is recomputed weekly, while the UnIT score remains invariant, representing a geospatial phenotype modulating transmission.

Our overall scheme is summarized in Fig 1. We leverage the county-specific incidence patterns observed for the past Influenza epidemics to compute the UnIT score, which then is used as a new fixed effect to infer a general linear model (GLM) for county-specific COVID-19 weekly count totals, alongside other putative covariates. The coefficients computed for the GLM model (stag 1, updated weekly) are then used to “correct” the COVID-19 count, replacing the observed count vector with the weighted linear combination of the socio-economic, demographic and the UnIT risk covariates. Intuitively, one may visualize this step as analogous to replacing a somewhat diffused set of observed points with a fitted line in linear regression. Finally, in stage 2 this corrected incidence vector is used to train ensemble regressors (updated weekly) that predict the next week’s county-specific count totals. Importantly, the GLM model in the first stage and the regressors in the second stage are updated weekly, while the UnIT score remains invariant. The key ingredient that makes simple ensemble regressors in the final step to perform better than more involved tools reported in the literature is the information-rich UnIT score, which potentially informs about complex transmission patterns modulating Influenza-like incidence native to each US county. Importantly, for each week, we compute one GLM model, and a fixed set of ensemble regressors, which are then used to predict case counts for individual counties, *i*.*e*., we do not have separate models for each county, and the county-specific effects are captured by the spatial variation of the putative covariates.

Our ability to leverage Influenza infection patterns to inform COVID-19 modeling is not surprising. COVID-19 and Influenza are both respiratory disorders, which present as a wide range of illnesses from asymptomatic or mild through to severe disease and possible death. Both viruses are transmitted by contact, droplets and fomites [23]. Current efforts to curb the spread of COVID-19 worldwide has also reduced Influenza cases [24–26]. However, to the best of our knowledge, the current paradigms have not capitalized on this similarity between the transmission mechanisms of the two viruses. This is not simply an oversight: an effective approach to leverage flu patterns in COVID-19 modeling is non-trivial. Despite similarities outlined above, there are important empirically observed differences between the two diseases precluding a “drop-in” replacement, *e*.*g*., COVID-19 has possibly a higher reproduction number [27–29], can be spread widely by asymptomatic carriers (more so than Influenza [30, 31]), is estimated to have a potentially higher mortality rate [32], is novel, *i*.*e*., is infecting a host population with almost non-existent immunity, and the COVID-19 pandemic has induced a global trend of social distancing policies alien to the seasonal flu dynamics. Despite these challenges, the UnIT score has significant predictive value, more than manual combinations of putative factors investigated so far.

## Results

**Fig 2:**
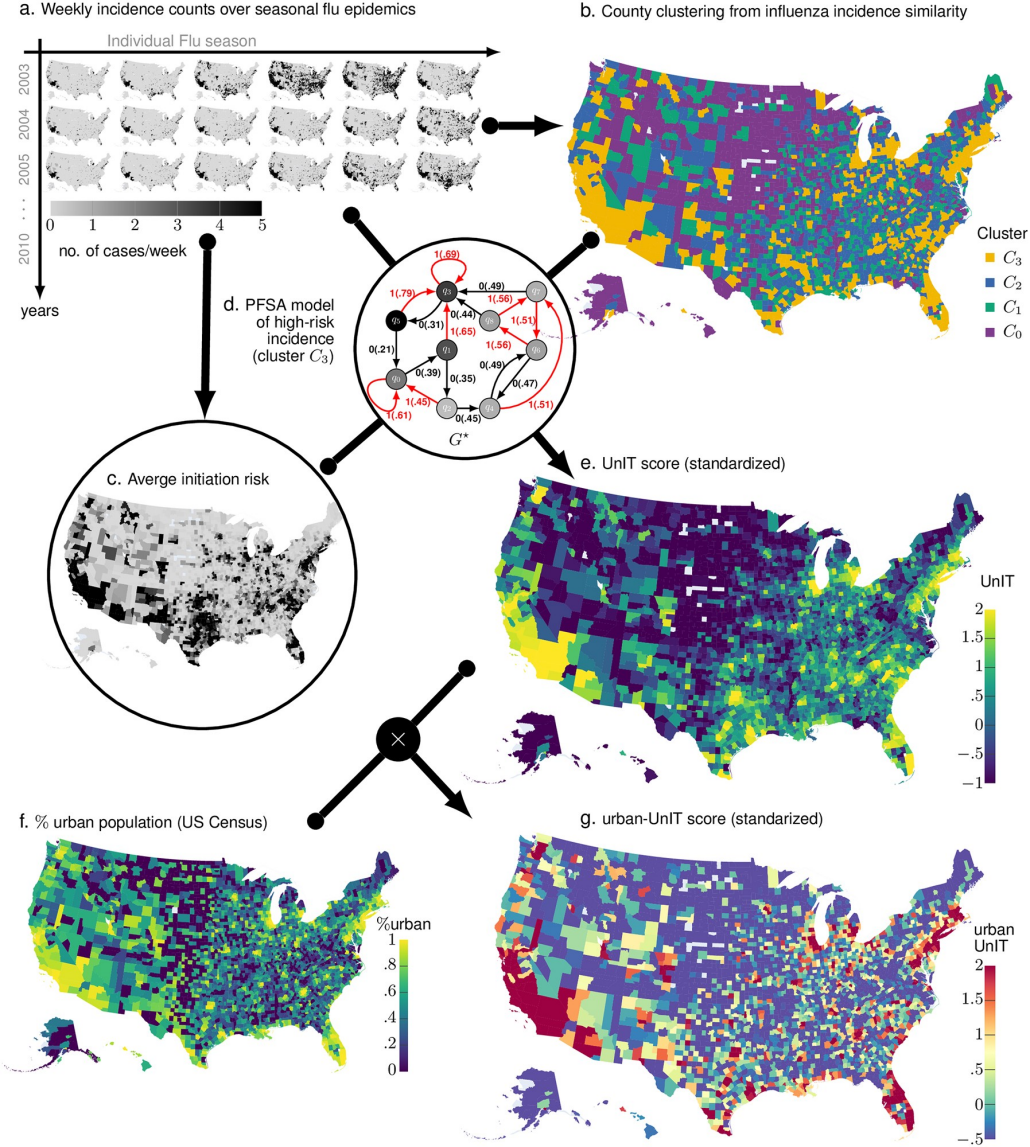
UnIT risk calculation. **Panel A**. Our approach begins with collecting weekly county-wise new case counts of the seasonal flu epidemic spanning Jan. 2003 to Dec. 2012 from a large national database of insurance claims records (Truven MarketScan). We identify weekly Influenza diagnoses using ICD codes related to influenza infection (See Materials and methods), and end up with county-specific integer-valued time series for each US county for each flu season. **Panel B**. These 471-week-long integer-valued time-series are used to compute pairwise similarity between the counties using our new approach of computing intrinsic similarity between stochastic sample paths (See (5)). This similarity matrix induces county clusters *C*_0_, *C*_1_, *C*_2_ and *C*_3_, inferred via standard spectral clustering. **Panel C**. The flu incidence time series allow us to identify counties which register cases in the first couple of weeks of each flu season. Averaged over all the seasons this gives us a measure of average epidemic initiation risk. **Panel D**. Using the incidence series for the county cluster with maximal average initiation risk we compute a specialized HMM model (PFSA, see Materials and methods) *G*^⋆^. **Panel E**. Then, we compute the UnIT risk phenotype of each county as the sequence likelihood divergence (SLD, See (8)) between the incidence sequence observed and the inferred PFSA model *G*^⋆^. **Panels F and G**. Finally, the urban-UnIT risk is computed by scaling up the UnIT risk with the fraction of urban population in each county, as obtained from US census (**Panel f**). We show that this risk phenotype is highly predictive of weekly case count of COVID-19, while only dependent on Influenza epidemic history.

In our results on COVID-19 modeling, in addition to the UnIT risk, we also use a scaled version of the UnIT risk, which we call the urban-UnIT risk (See Fig 2G). The urban-UnIT risk is the product of the estimated UnIT risk and the percentage of urban population in each county (See Materials and methods for details). To demonstrate the role of urban-UnIT risk as a meaningful risk phenotype of US counties, we first investigate its influence as a covariate driving the weekly total new case count for COVID-19. Diverse putative driving factors have been investigated to explain/model the epidemiological data emerging over the course of the current pandemic. Suspected factors include weather and pollution covariates [34], population density, socio-economic factors such as poverty, median household income, various measures of income inequality, and fraction of population without medical insurance, demographic variables such as the percentage of African-American, Hispanic and other minorities in the local population, percentage of population aged over 65 years, and gender [34–39]. A common approach here is the use of Poisson regression [40] to establish the statistical significance and relative magnitude of the influence of the various individual factors, and their suspected interactions. We identified the variables that have been repeatedly cited as the most important driving factors, and investigated the effect of adding in the urban-UnIT and the UnIT scores in multi-variate Poisson regression models, with weekly new case count total as the endogenous (response) variable.

Our first key result is that in our models, the urban-UnIT score significantly dominates typical putative factors. The UnIT score emerges as the second most important covariate (See Table 1). Since we standardize covariates to zero mean and unit variance, the magnitude of the inferred coefficients potentially reflect their relative impact in the models. This is illustrated in Table 1 where we show the inferred coefficients in a Poisson regression model with the typical covariates along with the urban-UnIT and UnIT risks. In Table 1, we consider county-specific COVID-19 case counts available on 2021–05-30, and note that the magnitude of the coefficient for urban-UnIT risk is close to being an order of magnitude larger than that for the next most influential covariate (0.849 for urban-UnIT risk vs −0.156 for % of population in poverty, and −0.127 median household income). Note that all coefficients inferred are strongly significant with *p* < 0.01.

**Table 1:** Inferred coefficients in multi-variate Poisson regression for putative factors driving weekly case totals as of 2021–05–30^⋆^.

	description	coef.	z-value	0.025	0.975
pop	total population	0.083	2333.322	0.083	0.083
%65+	percentage of population over 65 years old	−0.031	−117.370	−0.031	−0.030
%minority	percentage of minority (non-white) population	0.012	22.936	0.011	0.013
%black	percentage of black population	−0.032	−66.904	−0.033	−0.031
%Hispanic	percentage of Hispanic population	0.016	82.326	0.015	0.016
%poverty	percentage of population in poverty	−0.156	−356.559	−0.157	−0.155
income	median household income	−0.127	−427.632	−0.128	−0.127
%urban	percentage of urban population	0.101	134.806	0.099	0.102
UnIT	risk phenotype of US counties	0.317	445.914	0.316	0.318
urban-UnIT	UnIT-risk phenotype scaled up by %urban	0.849	934.880	0.848	0.851

^⋆^All *p*-values are < 0.0005.

**Fig 3:**
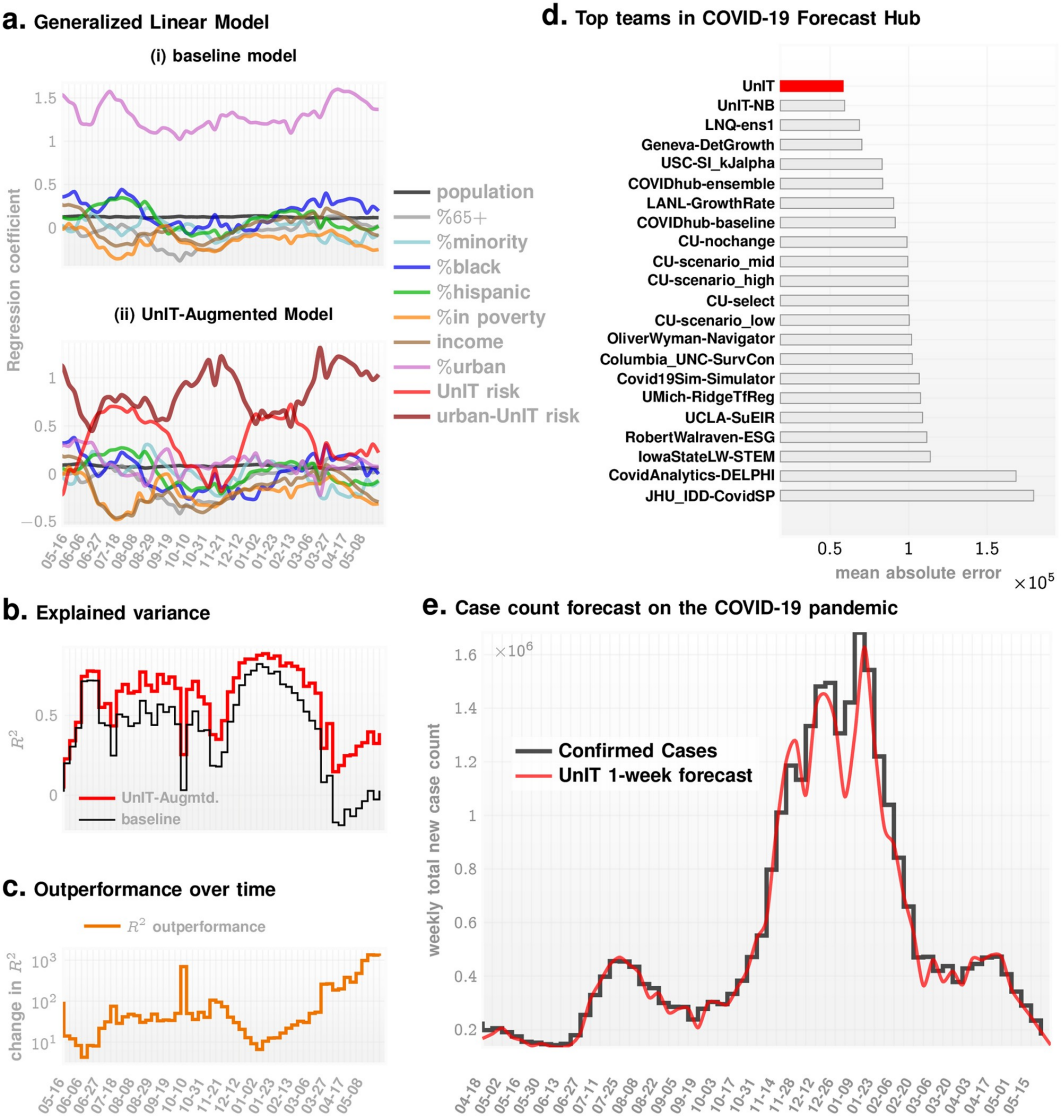
Results. **Panel A**. We compare the coefficients inferred in multi-variate Poisson regression for individual weeks of the COVID-19 pandemic for the range of covariates shown in the legend. We investigate two models: (i) the baseline model without the UnIT risk related covariates, and (ii) the model augmented with the UnIT risk (See (11)). We note that the urban-UnIT risk significantly dominates the remaining factors for the entire time-line of the pandemic. **Panel B**. The UnIT-augmented model has a significantly higher degree of explained variance as measured by *R*^2^. The percentage difference is shown in **panel C**, which demonstrates > 45% advantage for the major part of the pandemic time-line. **Panel D** illustrates that the UnIT-augmented approach achieves the smallest mean absolute error in one-week ahead county-wise incidence forecasts among the top performing teams from the COVID-19 ForecastHub Community. Finally, **panel E** illustrates the confirmed weekly total of case count summed over all counties vs our 1-week forecast.

Next to demonstrate the dominance of the UnIT risks throughout the current pandemic, we carry out the regression modeling at each week of the current pandemic. We find that urban-UnIT risk remains dominant over the entire pandemic time-line (See Fig 3A(ii)), by comparing 1) a baseline model with the covariates outlined in Table 1 with the exception of the two UnIT risk variables, vs 2) the full UnIT augmented model with all the enumerated covariates. The comparative results are shown in panels A(i) and A(ii) of Fig 3. Comparing the explained variance of the weekly confirmed case counts via the standard *R*^2^ measure (See Fig 3B and 3C), we note that the UnIT-augmented model has greater than significant advantage over the baseline model, explaining nearly 60% of the variance in the observed weekly COVID-19 case count totals (median *R*^2^ ≈ 0.651 for augmented model, and ≈ 0.448 for baseline model) for most of the pandemic time-line. Weekly inference of coefficients for the weeks between 2020-09-05 to 2021-01-23 is shown in Table 2.

**Table 2:** Inferred coefficients in multi-variate Poisson regression for individual weeks.

		pop	%65+	%minority	%black	%hispanic	%poverty	income	%urban	UnIT	urban-UnIT
**09–05**	***z*-value**	196.9	−79.8	26.2	−41.2	−26.1	−2.87	−67.1	28.6	63.7	59.7
	.025	0.079	−0.250	0.113	−0.189	−0.055	−0.021	−0.227	0.211	0.456	0.556
	.975	0.080	−0.238	0.132	−0.171	−0.048	−0.004	−0.214	0.242	0.485	0.594
	**coef**.	0.079	−0.244	0.122	−0.180	−0.051	−0.012	−0.221	0.226	0.471	0.575
**09–12**	***z*-value**	171.1	−90.6	13.0	−32.0	−63.6	−1.01	−51.3	29.9	52.5	54.6
	.025	0.080	−0.314	0.058	−0.165	−0.150	−0.014	−0.188	0.239	0.404	0.552
	.975	0.082	−0.301	0.078	−0.146	−0.141	0.005	−0.174	0.272	0.435	0.593
	**coef**.	0.081	−0.307	0.068	−0.155	−0.146	−0.005	−0.181	0.256	0.419	0.573
**09–19**	***z*-value**	199.4	−104.2	1.09	−26.3	−52.4	−26.0	−73.8	12.2	40.6	82.1
	.025	0.083	−0.333	−0.004	−0.136	−0.113	−0.126	−0.258	0.077	0.271	0.739
	.975	0.085	−0.321	0.016	−0.117	−0.105	−0.109	−0.244	0.106	0.298	0.776
	**coef**.	0.084	−0.327	0.006	−0.126	−0.109	−0.118	−0.251	0.091	0.285	0.757
**09–26**	***z*-value**	233.6	−132.9	−5.59	−25.0	−38.8	−50.5	−97.6	−9.54	33.4	103.1
	.025	0.088	−0.416	−0.038	−0.129	−0.080	−0.233	−0.339	−0.080	0.205	0.864
	.975	0.090	−0.404	−0.019	−0.110	−0.072	−0.215	−0.326	−0.052	0.230	0.898
	**coef**.	0.089	−0.410	−0.029	−0.120	−0.076	−0.224	−0.332	−0.066	0.217	0.881
**10–03**	***z*-value**	196.6	−106.6	7.95	−52.3	−62.5	−52.3	−97.6	6.37	13.5	111.3
	.025	0.081	−0.322	0.029	−0.251	−0.132	−0.247	−0.337	0.030	0.075	0.937
	.975	0.083	−0.311	0.048	−0.233	−0.124	−0.229	−0.323	0.057	0.101	0.970
	**coef**.	0.082	−0.317	0.038	−0.242	−0.128	−0.238	−0.330	0.043	0.088	0.953
**10–10**	***z*-value**	203.9	−122.3	6.95	−58.5	−87.2	−58.3	−107.9	12.0	14.2	114.6
	.025	0.083	−0.348	0.023	−0.262	−0.177	−0.258	−0.352	0.063	0.074	0.900
	.975	0.084	−0.337	0.040	−0.245	−0.169	−0.241	−0.340	0.088	0.097	0.932
	**coef**.	0.083	−0.343	0.031	−0.253	−0.173	−0.250	−0.346	0.075	0.086	0.916
**10–17**	***z*-value**	203.7	−108.1	−5.91	−39.9	−80.9	−76.4	−125.0	14.7	10.7	129.1
	.025	0.079	−0.280	−0.036	−0.184	−0.152	−0.321	−0.390	0.075	0.049	0.950
	.975	0.081	−0.270	−0.018	−0.166	−0.145	−0.305	−0.378	0.098	0.071	0.979
	**coef**.	0.080	−0.275	−0.027	−0.175	−0.149	−0.313	−0.384	0.087	0.060	0.964
**10–24**	***z*-value**	248.5	−127.6	−15.7	−44.0	−86.7	−86.2	−142.1	13.4	−9.64	160.3
	.025	0.083	−0.299	−0.075	−0.187	−0.145	−0.329	−0.403	0.060	−0.060	1.06
	.975	0.085	−0.290	−0.059	−0.171	−0.139	−0.314	−0.392	0.081	−0.039	1.09
	**coef**.	0.084	−0.294	−0.067	−0.179	−0.142	−0.321	−0.398	0.070	−0.050	1.08
**10–31**	***z*-value**	233.0	−136.3	−37.1	−35.0	−93.4	−93.4	−142.1	11.8	−28.2	189.8
	.025	0.076	−0.291	−0.164	−0.148	−0.144	−0.331	−0.367	0.047	−0.144	1.17
	.975	0.078	−0.283	−0.147	−0.132	−0.138	−0.317	−0.357	0.066	−0.125	1.19
	**coef**.	0.077	−0.287	−0.156	−0.140	−0.141	−0.324	−0.362	0.057	−0.135	1.18
**11–07**	***z*-value**	289.0	−165.7	−58.5	0.650	−80.6	−117.7	−147.6	26.7	14.5	176.5
	.025	0.079	−0.297	−0.224	−0.005	−0.103	−0.344	−0.313	0.102	0.050	0.917
	.975	0.081	−0.290	−0.209	0.009	−0.098	−0.333	−0.305	0.118	0.065	0.937
	**coef**.	0.080	−0.293	−0.217	0.002	−0.101	−0.338	−0.309	0.110	0.058	0.927
**11–14**	***z*-value**	341.9	−190.4	−69.7	−45.7	−173.6	−113.6	−163.5	29.2	−49.2	258.1
	.025	0.082	−0.301	−0.230	−0.146	−0.206	−0.301	−0.305	0.099	−0.186	1.21
	.975	0.083	−0.294	−0.218	−0.134	−0.201	−0.291	−0.298	0.113	−0.171	1.23
	**coef**.	0.083	−0.297	−0.224	−0.140	−0.203	−0.296	−0.301	0.106	−0.178	1.22
**11–21**	***z*-value**	392.9	−149.3	−59.8	−44.7	−138.5	−101.1	−155.5	41.6	−23.5	253.1
	.025	0.083	−0.213	−0.178	−0.128	−0.147	−0.244	−0.263	0.135	−0.086	1.10
	.975	0.084	−0.207	−0.166	−0.117	−0.143	−0.234	−0.257	0.149	−0.073	1.12
	**coef**.	0.084	−0.210	−0.172	−0.122	−0.145	−0.239	−0.260	0.142	−0.080	1.11
**11–28**	***z*-value**	394.9	−101.7	−14.4	−98.3	−129.4	−82.0	−142.9	49.1	1.77	230.3
	.025	0.082	−0.146	−0.044	−0.259	−0.141	−0.202	−0.242	0.170	−0.001	1.05
	.975	0.083	−0.140	−0.033	−0.249	−0.137	−0.193	−0.235	0.184	0.013	1.07
	**coef**.	0.083	−0.143	−0.038	−0.254	−0.139	−0.197	−0.238	0.177	0.006	1.06
**12–05**	***z*-value**	496.9	−90.0	−25.4	−62.6	−93.1	−87.5	−121.5	40.2	46.9	217.9
	.025	0.089	−0.120	−0.070	−0.158	−0.094	−0.199	−0.187	0.132	0.150	0.931
	.975	0.090	−0.115	−0.060	−0.148	−0.090	−0.190	−0.182	0.145	0.163	0.948
	**coef**.	0.090	−0.118	−0.065	−0.153	−0.092	−0.195	−0.184	0.139	0.156	0.939
**12–12**	***z*-value**	591.2	−51.8	32.5	−112.2	−77.1	−72.2	−113.2	53.3	107.2	180.7
	.025	0.094	−0.067	0.067	−0.245	−0.074	−0.156	−0.162	0.177	0.344	0.757
	.975	0.095	−0.062	0.076	−0.237	−0.071	−0.147	−0.157	0.190	0.357	0.773
	**coef**.	0.094	−0.064	0.072	−0.241	−0.073	−0.151	−0.160	0.184	0.351	0.765
**12–19**	***z*-value**	647.8	−28.0	66.6	−123.6	13.7	−80.1	−113.3	37.6	158.3	150.9
	.025	0.095	−0.037	0.139	−0.264	0.011	−0.171	−0.162	0.128	0.524	0.644
	.975	0.096	−0.032	0.147	−0.255	0.014	−0.163	−0.157	0.142	0.537	0.661
	**coef**.	0.096	−0.035	0.143	−0.259	0.013	−0.167	−0.159	0.135	0.531	0.653
**12–26**	***z*-value**	640.5	7.58	83.1	−116.2	32.2	−61.2	−79.0	54.9	168.4	109.6
	.025	0.099	0.007	0.181	−0.258	0.030	−0.140	−0.119	0.210	0.615	0.513
	.975	0.099	0.013	0.190	−0.250	0.033	−0.132	−0.113	0.226	0.630	0.532
	**coef**.	0.099	0.010	0.185	−0.254	0.032	−0.136	−0.116	0.218	0.623	0.522
**01–02**	***z*-value**	654.5	−0.500	44.2	−60.8	44.1	−73.4	−73.6	21.3	161.9	145.8
	.025	0.097	−0.003	0.097	−0.139	0.040	−0.162	−0.108	0.073	0.560	0.647
	.975	0.098	0.002	0.106	−0.130	0.043	−0.153	−0.102	0.088	0.574	0.665
	**coef**.	0.098	−0.001	0.102	−0.135	0.042	−0.157	−0.105	0.080	0.567	0.656
**01–09**	***z*-value**	662.6	11.2	62.5	−78.4	44.6	−80.1	−86.6	39.3	188.0	143.3
	.025	0.093	0.011	0.125	−0.160	0.037	−0.160	−0.115	0.129	0.596	0.584
	.975	0.094	0.015	0.133	−0.152	0.040	−0.153	−0.110	0.142	0.609	0.600
	**coef**.	0.094	0.013	0.129	−0.156	0.038	−0.157	−0.113	0.136	0.602	0.592
**01–16**	***z*-value**	624.0	7.83	58.8	−50.7	102.1	−79.5	−67.9	25.7	171.7	143.6
	.025	0.090	0.007	0.124	−0.110	0.089	−0.165	−0.094	0.089	0.590	0.628
	.975	0.091	0.012	0.133	−0.102	0.092	−0.158	−0.089	0.103	0.604	0.645
	**coef**.	0.090	0.010	0.128	−0.106	0.090	−0.162	−0.091	0.096	0.597	0.637
**01–23**	***z*-value**	495.0	14.9	21.4	6.71	112.2	−78.9	−48.1	22.6	149.7	129.2
	.025	0.085	0.018	0.050	0.012	0.108	−0.183	−0.075	0.085	0.570	0.627
	.975	0.085	0.023	0.060	0.021	0.112	−0.174	−0.069	0.102	0.585	0.647
	**coef**.	0.085	0.020	0.055	0.016	0.110	−0.178	−0.072	0.093	0.577	0.637

Coefficients with *p*-value in [0.01, 0.05) are colored blue, and those with *p*-value ≥ 0.05, red. All other *p*-values are < 0.01.

**Fig 4:**
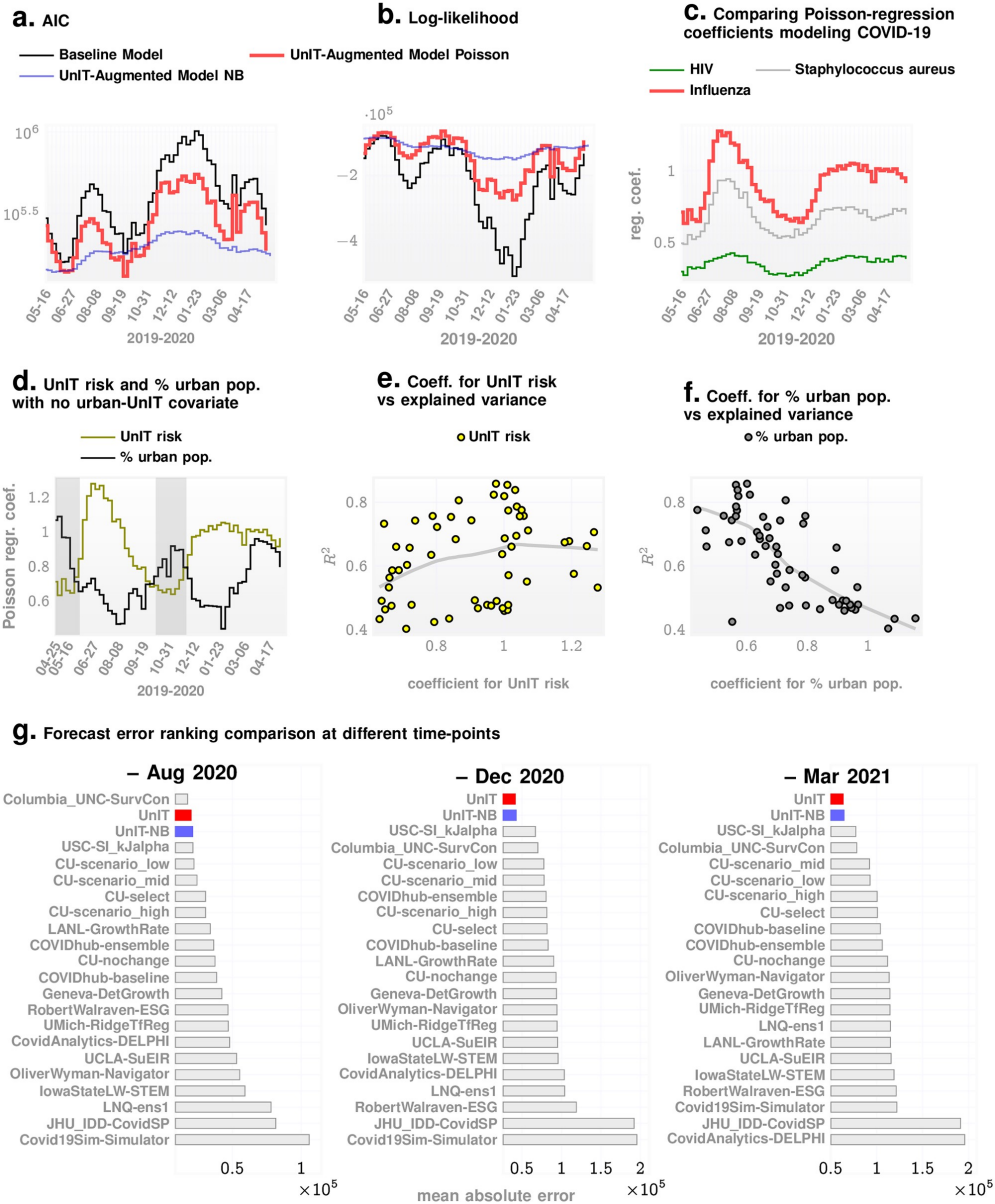
More results. **Panel A**. AIC over time (lower is better) for the baseline model, and the UnIT-augmented models with Poisson and and negative binomial (NB) regressions in the first stage respectively. The NB-based approach has lower AIC on average. Similar conclusion is reached in **panel B** considering the log-likelihood of the models over time (higher is better). The Poisson-approach (red) ultimately makes slightly better predictions from the two stage modeling, as shown in the bottom row of the figure. **Panel C** illustrates that influenza is a good choice for a COVID-19-similar disease, producing the largest coefficients for the risk variable among bacterial infections such as Staphylococcus aureus (which is worse than Influenza), or chronic infections such as HIV (which is still worse). **Panel D**. shows that temporal variation of the regression coefficients for UnIT risk and % of urban population. Here we used Poisson regression leaving out the urban-UnIT risk covariate in the augmented model to highlight the role of UnIT risk vs % urban population: except in the shaded periods, the coefficient for the UnIT risk dominates. **Panel E** and **panel F** show the variation of the coefficients for the UnIT risk and % urban population with adjusted *R*^2^. We note that the LOWESS fit shows that *R*^2^ increases and saturates as the coefficient for UnIT risk increases, whereas it drops rapidly with increasing values of the coefficient for % urban population. This suggests that when the covariate for the % of urban population is more important, our explained variance is low. **panel G** illustrates the mean absolute forecast errors at different points in the pandemic, highlighting the results obtained with Poisson and NB regressions (See also S1 Fig).

We note that simply comparing the magnitude of the regression coefficients between the baseline and the UnIT augmented models might not justify the claim that our new covariates improve the model. To establish this, we compute the Akaike Information Criteria (AIC, the lower the better) [41], and the model log-likelihood measures (the higher the better) over time as shown in Fig 4A and 4B. We note that the augmented model is clearly dominating the baseline for both measures. An second issue is the justification for assuming that our response variable follows a Poisson distribution. Poisson regression makes strong assumptions about the dispersion characteristics of the data, in particular, that the mean and the variance of the response variable is identical. If our data is significantly overdispersed, then negative binomial (NB) regression is generally suggested to be a better choice, which lacks this particular constraint. We find that our data is indeed somewhat overdispersed (as determined by calculating the ratio of deviance to the residual degree of freedom [42], which turns out to be > 1). We compare the effect of replacing the Poisson regression with NB regression in the first stage of our approach, which results in similar, but somewhat worse predictive performance of the case counts at the end of our predictive pipeline. We see that the NB-model has on average lower AIC and higher log-likelihood compared to the Poisson model (See Fig 4A and 4B), but nevertheless the final performances are slightly worse with the NB model (See Fig 4G and S1 Fig). Hence, we decided to use the Poisson regression over NB in the first stage of our approach. It is important to note that we are only using the Poisson regression in the first stage to generate features that get used by the ensemble regressor in the second stage. We are not using the first stage model for prediction directly, and not using any results that require residual normality or the estimation of confidence bounds, and hence are not significantly affected by errors resulting from overdispersion.

A third issue lies with the claim that the regression coefficient for urban-UnIT risk dominates the other covariates in the augmented model. Since the covariate for the % of urban population dominates in the baseline model, one might argue that the dominant behavior of the urban-UnIT risk purely arises from its definition: the product of the % of urban population with the UnIT risk. Also, comparing Fig 3A and 3B, it appears that the urban-UnIT risk is anti-correlated with the explained variance *R*^2^, which might undermine the claim that this new covariate improves our model. To investigate these concerns, we investigated a separate model where we only included the UnIT risk (and not the urban-UnIT risk)), and compared the coefficients for the UnIT risk and the % of urban population. The results are shown in Fig 4D, 4E and 4F. Panel d shows that the UnIT risk dominates the % of urban population on average, and over the timeline of the pandemic, except within some limited periods. Additionally, a locally weighted scatterplot smoothing (LOWESS) fit [43] in panels E and F show that while the explained variance increases (and saturates) with increasing values of the coefficient for UnIT risk, it rapidly falls with increasing values of the coefficient for % of urban population. This establishes that the UnIT risk does have more explanatory information, and the behavior of urban-UnIT risk may be attributed to the decreasing predictability of the response variable as the effect of % of the urban population becomes more important. As for the comparison between the augmented and the baseline models with respect to the dominance of the % of urban population, it is expected that urban population (cities) matter, with infection spreading more easily among people living in close contact, explaining why this covariate is dominant in the baseline model. However, combined with the UnIT risk, we get a more predictive covariate (the urban-UnIT risk), which then dominates in the augmented model.

In addition to the above considerations, we demonstrate the robustness of the UnIT score via multiple modes of perturbation, namely by 1) deleting the top 10% of the counties ranked by the highest number of COVID-19 cases per capita, and 2) randomly selecting only 75% of the counties to include in the analysis. Under all such perturbations, the UnIT score retains its position as the dominant explanatory factor (See S3 Fig).

**Fig 5:**
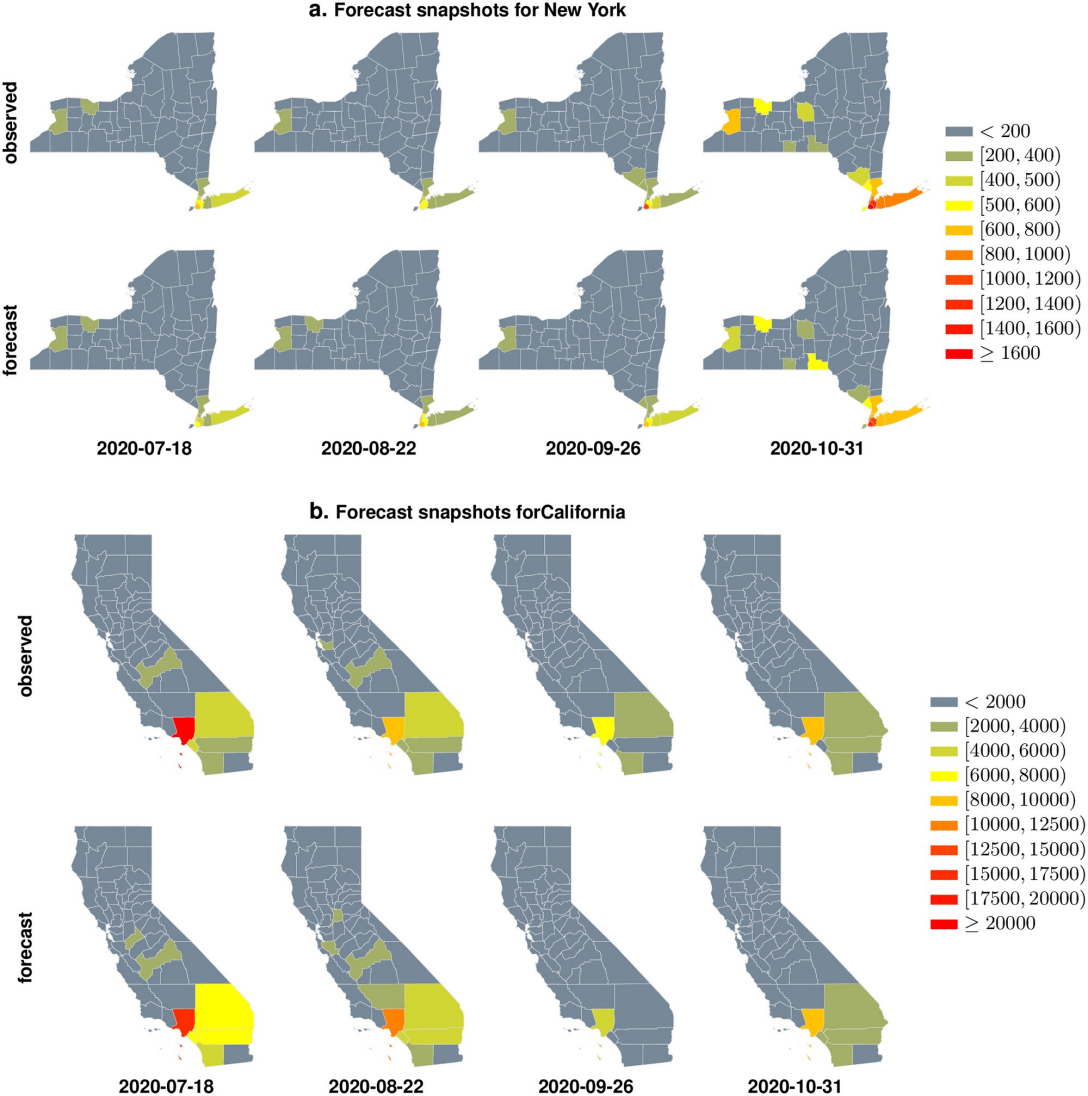
**Panel a**. We compare our forecasts of weekly case counts (1 week ahead forecasts) with observed confirmed cases on counties from the state of New York. **Panel b**. We compare the weekly forecasts with observed count for the state of California. We note that in both states, for the weeks included in this limited snapshot, the predicted count matches up well with what is ultimately observed. The cartography in this figure is generated from scratch using opensource shape files available at https://www.sciencebase.gov/catalog/item/581d051de4b08da350d523cc using GeoPandas [33].

Finally, we investigate our ability to forecast weekly COVID-19 case count totals across the US counties. Using a simple forecast model (See Eq (3a) in Materials and Methods) that incorporates the UnIT risk we outperform the state of the art models from the US COVID-19 modeling community (https://covid19forecasthub.org/community), achieving the least mean absolute error in 1-week ahead county-specific incidence forecasts (See Fig 3D and 3E) over the entire pandemic time-line. The predicted and confirmed case counts for New York and California are shown at selected weeks over the pandemic, where our 1-week forecasts match up well with the observed counts (See Fig 5) in these two US states hit hard by the COVID-19 pandemic.

## Discussion

The global modeling community responded to the COVID-19 pandemic with diverse tools [17, 44–47] to predict case counts, COVID-19-related hospitalizations and deaths (See Table A in S1 Text for an incomplete list). The proposed approaches range from county-level meta-population estimates to stochastic compartmental models to fitting Gaussian processes to raw data to survival-convolution models to growth rate dynamics to models that take into account human mobility and social distancing policies explicitly. In the US, predictions from individual contributing groups are been used to inform an ensemble forecast [48], which is currently live at a web-based visualization portal at https://viz.covid19forecasthub.org/ (the COVID-19 forecasthub). As a contribution to this community, we report a precise yet simple model for forecasting case counts; one that operates without explicit social distancing and other hard-to-measure parameters, yet outperforms the operating models at the COVID-19 forecasthub, including the ensemble forecast. Our current 1-week forecast may be viewed at the COVID-19 forecasthub webpage (team: UChicagoCHATTOPADHYAY-UnIT), and complete software with usage instructions (See Software Usage in S1 Text) is publicly available at https://github.com/zeroknowledgediscovery/unitcov.

In addition to the development of forecasting tools, general epidemiological modeling of COVID-19 has progressed in two broad categories: 1) deep theoretical approaches to understand disease propagation in epidemics extending classical compartmental models or their variations [17, 44–47]. These investigations aim to estimate the theoretical reproduction number of COVID-19, and other epidemiological quantities associated with the virus. And, 2) in the second category, studies have focused on identifying putative factors driving the differential severity and case counts across regions, demographic strata and age groups [34–39, 49–51]. The first category of studies may be seen as theoretical epidemic modeling, and the second as inferential analyses [52], *i*.*e*., infer how nature associates responses with input variables aiming to work out the differential impact of putative factors. The current study improves results in the second category by presenting the UnIT score as a highly explanatory covariate, and then demonstrates its ability to make precise incidence forecasts.

The UnIT risk exposure of a US county is conceived of as intrinsic similarity of the time series of weekly total of new flu cases to that observed in counties at high risk of an epidemic initiation. Thus, central to our approach is the notion of intrinsic similarity between stochastic processes, particularly if the structure of the underlying processes is unknown. Such (dis)similarity is quantified by the notion of sequence likelihood divergence (SLD), which lies at the heart of our computation (See Eq (8) in Materials and Methods). SLD is a generalization of the notion of divergence of probability distributions (KL divergence [53]) to potentially non-iid stochastic processes. Similar to how we quantify the deviation of a probability distribution *p* from *q* by their KL-divergence D(p∥q), SLD measures the divergence of a stochastic process *P* from *Q* as D(P∥Q). The actual computations are distinct despite the identical notation used (See *Intuitive Example* in Materials and methods). Additionally, the log-likelihood of a sample path *x* being generated by a process *G*, denoted as *L*(*x*, *G*), converges in probability:

$$\begin{array}{c} L(x,G)\to H(X)+D(X∥G) \end{array}$$ (1)

with increasing length of *x*, where *X* is the true generator of the sample path *x*, and *H*(⋅) is the entropy rate [53] function (See Materials and methods, Theorem 1). Importantly, if the processes are modeled by a special class of Hidden Markov Models known as Probabilistic Finite State Automata (PFSA) [54], then the estimation of the LHS of Eq (1) becomes tractable (Algorithm A in S1 Text). Using SLD we can efficiently compute the dissimilarity between two observed sample paths, estimated as the deviation between the underlying generators.

Thus, the UnIT risk (denoted as *ν*) of a county is defined as the SLD between the underlying process driving incidence counts and a high risk process initiating the epidemic. Since these processes are hidden, and only sample paths are observable, we formulate an estimator for the UnIT risk as follows: we begin with weekly county-wise confirmed case counts of the seasonal flu epidemic spanning nearly a decade (nine flu seasons between 2003–2012, See Fig 2A). These are obtained by looking for Influenza related diagnostic codes reported in each week in each county in the Truven Marketscan insurance claims database [55]. This database consists of over 150 million patients *i*.*e*. almost a third of the US population, and despite limitations (under-reporting of non-severe influenza cases, and reporting/coding uncertainties), provide a detailed record of flu season incidence dynamics. These relatively short integer-valued time-series (each spanning 471 weeks) are used to compute pairwise similarity between the counties (using the SLD-based approach, see Materials and methods), which then induces a partition of the 3094 US counties into a pre-specified number of clusters, obtained by using standard clustering techniques, *e*.*g*. spectral clustering [56] (See Fig 2B). We note here that the number of clusters (four) is chosen via standard heuristic considerations [57], and increasing this number somewhat does not significantly impact our results. With these county-clusters in hand, we next inspect the initial weeks of the nine flu seasons to estimate the empirical probability of a specific county reporting cases within the first couple of weeks of a flu season | these counties are at high initiation risk empirically (See Fig 2C). We find that one specific cluster accounts for almost all of the counties at high risk of flu season initiation. Focusing on the set of counties in this high risk cluster, we infer [54] a PFSA *G*^⋆^, assuming that the incidence series at each of these counties is a sample path from the same underlying stochastic process (See Fig 2D). This is a simplification, aimed at obtaining an average model driving the incidence dynamics at initiation, ignoring the variation in the structure and parameters of the underlying processes among the high risk counties themselves. Finally, we estimate the UnIT risk exposure of each county with count sequence *x* as:

$$\begin{array}{c} \hat{\nu (x)}≜L\left(x,G^{⋆}\right)-\hat{H(X)}\to D(X∥G^{⋆}) \end{array}$$ (2)

where the convergence to the divergence between the local process *X* and the inferred high risk process *G*^⋆^ occurs in probability as length of *x* increases.

To carry out this computation, we need a consistent estimate [58] of the entropy rate of the process *X* from *x*. This is non-trivial [59, 60] if *X* is not an iid process. We may either: 1) estimate the entropy rate from the observed sample path [61], or 2) compute an upper bound of the entropy rate assuming *X* is iid for the purpose of computing *H*(*X*) only. The second approach is computationally simpler, but only allows us to estimate a lower bound of the UnIT risk. For simplicity we present results with only the second approach (See Fig 2E), *i*.*e*. using a lower bound to the UnIT risk, which is nevertheless demonstrated to have significant predictive value, especially when scaled up with the percent of the county-specific urban population.

The estimated urban-UnIT risk obtained by scaling the UnIT risk with the fraction of urban population is then used to verify its dominant explanatory role amongst suspected covariates as discussed before. Finally this risk phenotype is used to make weekly case count forecasts, one week ahead of time, on a per county basis. The forecast model (See Eq (3a) in Materials and Methods) is simple; essentially an ensemble regressor with the urban-UnIT risk as an input feature, along with the previous week’s county-wise count totals, which as shown in Fig 3, outperforms more complex state of the art approaches.

It is important to consider how the performance of our algorithm varies along the pandemic timeline, particularly how it performs at the early days of the pandemic, and in the aftermath of peak infection. We plot the percentage forecast errors in panel B in S1 Fig, which shows that we underestimate the counts somewhat at the beginning of the pandemic, and overestimate somewhat post peak infection. While these trends might indicate that the effectiveness of the UnIT risk itself varies across the pandemic timeline, reporting inaccuracies might also be a contributing factor. Indeed, when we applied our approach to forecasting case counts using estimates of the true total infection as computed and posted at https://covid19-projections.com/infections/summary-counties/ [62, 63], these trends disappeared (See S2B and S2C Fig), suggesting the possibility that the official county-specific case count reports might have been underestimated in the early days, and are being overestimated somewhat after the infection peak in the United States. Limited access to COVID-19 tests in the early days of the pandemic supports these observations.

Additionally, comparing our forecasts against the reported case count (Fig 3E), it appears that the forecasts were worse around the peak infection point. However, the % forecast errors in S1 Fig confirm that there is no systematic uptick around the peak itself, and the larger differences visible in Fig 3E is due to scaling up of a similar error fraction as the case count exploded in the United States.

With the UnIT risk appearing to have usable information that helps us predict the counts better, it is important to ask if we could have used the incidence patterns of other diseases, either in conjunction to the UnIT risk or by themselves to improve the forecasts. We investigated the applicability of observed incidence dynamics of other infectious agents such as that of common bacteria *e*.*g*. Staphylococcus aureus (Staph) or viruses causing chronic infections such as the Human Immunodeficiency virus (HIV). As shown in Fig 4C, the Poisson regression coefficients for influenza dominate the others over time, with HIV significantly worse compared to Staph. This is not surprising, since bacterial infections as well as HIV are spread by mechanisms very different from that of COVID-19. More detailed investigations in this direction will be taken up in future, with a principled mechanism to characterize the epidemiological “similarity” of different infections from observed incidence patterns.

A second important question is the generalizability of our proposed approach to other infections, epidemics, and in other geographical regions. As a demonstration, we apply our approach to the prediction of HIV incidence in the year 2011, using influenza to construct the risk covariate as before (data restricted upto 2010). The results are shown in S2 Fig. The predictions are not very useful, as expected. In addition to influenza being not “similar” to HIV, the latter is a chronic infection, taking generally longer to serroconvert (≈ 2 months [64]), making weekly predictions not particularly appropriate. This suggests that to apply our approach for diseases other than COVID-19, we would need to identify a pathogen that transmits via similar mechanisms as that of the target organism, and also one for which we have geospatial incidence data at our disposal. Application of this approach beyond United States would require detailed incidence data on influenza and COVID-19. Our current access to the electronic administrative is currently limited to the United States, which limits our current validation to the US. Future work will attempt to test applicability in other regions of the world.

### Limitations & conclusion

A source of uncertainty in our approach is the use of diagnostic codes from insurance claims to infer seasonal flu incidence. Influenza is in general hard to track, since less severe cases are seldom reported. Additionally, electronic health records are also inherently noisy, and suffers from potential coding errors by physicians, and other artifacts. Similarly the number of confirmed COVID-19 cases is also a function of how many tests are actually administered, and the fraction of the infected population who are asymptomatic. Thus, we are forecasting the number of detected cases as opposed to true disease incidence.

Additionally, our database of diagnostic codes needs to have geospatial metadata, *i*.*e*, we need to know the county in which each patient was located at the time they contracted influenza. This information has been redacted recently from the Truven database due to privacy concerns, implying that we could only use data upto 2011 to construct the UnIT risk. Leaving out random years one at a time from this 9 year period did not affect the results significantly, suggesting that the key patterns we are leveraging do not change too fast. The effect of considering newer data from other sources, will be investigated in future.

An important limitation in our modeling are current assumptions on the distribution of the response variable. While we achieved good prediction results, future work in this direction might consider application of recently reported strategies that deal with non-Poisson or non-Gaussian distributions [65] to further refine our models.

Importantly our results do not imply that Influenza and COVID-19 are similar in their clinical progression. Indeed, a limitation of our approach is its reduced ability to predict COVID-19-related deaths (S4 Fig and Tables B and C in S1 Text). Our death count forecasts are worse than the top few contributors [66] to the COVID-19 forecasthub. We hypothesize that this reduced effectiveness is attributable to differences in the clinical progression of Influenza and COVID-19: COVID-19 is a more serious disease, and while historical flu patterns may be leveraged to predict the number of cases, performance suffers when we attempt to extend the same strategy to predict the mortality.

In this study we demonstrate that leveraging the knowledge of the incidence fluctuations in one epidemic informs another with a broadly similar transmission mechanism, despite differences in the epidemiological parameters and the disease processes. The COVID-19 pandemic has highlighted the need for tools to forecast case counts early in the course of future pandemics, when only sparse data is available to train upon, by leveraging incidence pertaining to different epidemics of the past.

## Materials and methods

We begin by describing the forecast model, followed by the mathematical details underlying the risk measure itself.

### Forecast model

The UnIT score (*ν*) is a spatially varying time-invariant measure. Thus, to forecast temporal changes in weekly incidence we consider the past week’s case count as a feature in training regressors as follows (where *X*_*t*_ is the observed case count at time *t*, and ${\hat{X}}_{t}$ is the forecast made for *t* at time *t* − 1):

$$\begin{array}{cc} \text{UnIT}\;\text{risk}\;\text{correction}\;\nu \;\text{and}\;\text{train}\;\text{GLM}\;g_{t} & X_{t}^{⋆}=g_{t}\left(X_{t},\nu ,v_{1},\cdots ,v_{m}\right) \end{array}$$ (3a)

$$\begin{array}{cc} \text{Train}\;\text{regressor}\;h_{t} & X_{t}=h_{t}\left(X_{t-1},X_{t-2}^{⋆},X_{t-1}^{⋆}\right) \end{array}$$ (3b)

$$\begin{array}{cc} \text{Forecasting}\;\text{estimate} & {\hat{X}}_{t+1}=h_{t}\left(X_{t},X_{t-1}^{⋆},X_{t}^{⋆}\right) \end{array}$$ (3c)

Here *g*_*t*_ is the generalized multivariate regression model (GLM) which carries out the Poisson regression, fitted with *X*_*t*_ as the target variable, and *ν*, *v*_1_, ⋯, *v*_*m*_ as exogenous variables, with a logarithmic link function (See (11)). *ν* is the urban-UnIT risk, and the rest of the variables *v*_1_, ⋯, *v*_*m*_ (as described in Table 1) are total population, fraction of population over 65 years, fraction of minorities in the population, fraction of Hispanics, fraction of the population reported as African-American or black, fraction of the population designated to be poor, and the median household income. Including the fraction of population living in urban environments as a separate variable does not change results significantly. In Eq 3b $X_{t}^{⋆}$ is the estimate of *X*_*t*_ obtained using the inferred coefficients in *g*_*t*_, and may be viewed as the noise corrected version of the current case count. Finally, we train a standard regressor between the corrected case count and the count observed in the next time step, and use it for forecasting one-week futures (Eq 3c). The choice of the specific regressor (random forest, gradient boosting, feed-forward neural networks or more complex variants) does not significantly alter our performance.

This is an exceedingly simple model compared to the approaches described in the literature, and is essentially a ensemble regressor with the UnIT-corrected case count as one of the features/inputs. Nevertheless we outperform the top state of the art models put forward by the COVID-19 modeling community (https://covid19forecasthub.org/community) in mean absolute error in county-specific incidence count estimates (See Fig 3D). As examples we illustrate the county-wise predicted and confirmed case counts for New York and California at selected weeks over the pandemic, which shows that our 1-week forecasts match up well with the counts ultimately observed (See Fig 5).

### Computing similarity from sample paths

Efficiently contrasting and condomness cannot be ignored. For such learning to occur, we need to define either a measure of deviation or, more genmparing stochastic processes is the key to analyzing time-dependency in epidemiological patterns, particularly where raerally, a measure of similarity to compare stochastic time series. Examples of such similarity measures from the literature include the classical *l*_*p*_ distances and *l*_*p*_ distances with dimensionality reduction [67], the short time series distance (STS) [68], which takes into account of irregularity in sampling rates, the edit based distances [69] with generalizations to continuous sequences [70], and the dynamic time warping (DTW) [71], which is used extensively in the speech recognition community.

A key challenge in the existing techniques is differentiating complex stochastic processes with subtle variations in their generative structures and parameters. When presented with finite sample paths from non-trivial stochastic processes, the state-of-the-art techniques often focus on their point-wise distance, instead of intrinsic differences in their (potentially hidden) generating processes. Our approach addresses this issue and demonstrably differentiates data streams indistinguishable by state-of-the-art algorithms.

Our intuition follows from a basic result in information theory: if we know the true distribution **p** of a random variable, we could construct a code [53] with average description length *h*(**p**), where *h*(⋅) is the entropy of a distribution. If we used this code to encode a random variable with distribution **q**, we would need h(p)+D(p∥q) bits on average to describe the random variable. Thus, deviation in the distributions show up as an additional contribution from the KL divergence term D(·∥·). Generalizing the notion of KL divergence to processes, we can therefore quantify deviations in dynamics via an increase in the entropy rate by the corresponding divergence.

### Intuitive example

As a more concrete example, consider sequences of length *n* generated by two iid processes $P_{1}=B(.5)$ and $P_{2}=B(.8)$, where *B*(*p*) is the Bernoulli process with parameter *p* [72]. Our objective is to estimate deviations in the binary sample paths generated by these processes. Here we choose iid processes for simplicity, which is *not a restriction in general for our approach*. Let us generate sequences of length *n* and use *E*_*ij*_ to denote the expected Hamming distance [73] between sequences generated by $P_{i}$ and $P_{j}$. It is easy to show that *E*_11_ = *E*_12_ = *E*_21_ = 0.5*n*, which implies that two sequences both generated by *B*(.5) are *not* more alike than two sequences where one is generated by *B*(.5) and the other by *B*(.8). Denoting:

$$\begin{array}{c} h_{1}=h\left(\left[.5,.5\right]\right)=1,h_{2}=h\left(\left[.8,.2\right]\right)=0.72,\\ d_{12}=D_{\text{kl}}(\left[.5,.5\right]\;∥\;[.8,.2])=0.32,\\ d_{21}=D_{\text{kl}}(\left[.8,.2\right]\;∥\;[.5,.5])=0.28, \end{array}$$

and letting *L*(*x*, *B*(*p*)) denote the log-likelihood of *B*(*p*) generating *x*, we define:

$$\begin{array}{c} v_{x}=[L(x,B(.5)),L(x,B(.8))] \end{array}$$ (4)

Then, by law of large numbers [74], we have:

$$\begin{array}{c} v_{x}\to \{\begin{array}{cc} \left(h_{1},h_{1}+d_{12}\right)=\left(1.0,1.32\right) & \text{if}\;x\;\text{is}\;\text{generated}\;\text{by}\;B(.5),\\ \left(h_{2}+d_{21},h_{2}\right)=\left(1.0,0.72\right) & \text{if}\;x\;\text{is}\;\text{generated}\;\text{by}\;B(.8). \end{array} \end{array}$$

which now clearly disambiguates the two processes indistinguishable by their expected Hamming distance, and the correct generator may be identified readily as the one corresponding to the index of the smaller entry in **v**_*x*_. Our approach generalizes this idea to more complex processes, where we cannot make the iid assumption a priori, thus necessitating the generalization of KL divergence from distributions to stochastic processes.

### Log-likelihood of generating sample paths

In the example above, the generating models are used to evaluate log-likelihoods, which are not directly accessible in our target application. The computation of the log-likelihood *L*(*x*, *G*) of a sequence *x* generated by a process *G*, is simple (See Algorithm A in S1 Text) if we restrict our stochastic processes to those generated by Probabilistic Finite State Automata (PFSA) [75–78]. PFSA are semantically succinct and can model discrete-valued stochastic processes of any finite Markov order, and can approximate arbitrary Hidden Markov Models [76] (HMM). Importantly, PFSA model finite valued processes taking values in a finite pre-specified alphabet. Thus, continuous or integer valued inputs must be quantized, in a manner described later.

In the context of the above discussion, we define dissimilarity Θ between observed sequences *x*, *y* as:

$$\begin{array}{c} \Theta \left(x,y\right)=\sum_{G^{i}\in G}\left|L\left(x,G^{i}\right)-L\left(y,G^{i}\right)\right| \end{array}$$ (5)

where $G^{i}\in G$ is a set of pre-specified PFSA generators on the same alphabet. And using PFSAs for our base models implies that this measure is easily computatble via multiple applications of Algorithm A in S1 Text for pseudocode of algorithm). In our approach, we use the set of four PFSA models shown in S5 Fig as G. Using a different set of models, which generate processes that are sufficiently pairwise distinct, does not significantly alter our results. These particular “base” models are chosen randomly from all possible PFSAs (See next section) with a maximum of 4 states. For a finite number of base models, Eq (5) does not technically yield a metric. However, one can approach a metric by increasing the number of models included in the base set. S5 Fig illustrates a comparison of this approach of comparing time series with the state of the art Dynamic Time Warp (DTW) algorithm. In particular, our approach is significantly faster yet produces a higher separation ratio (ratio of the mean distance between clusters computed by the two algorithms) for the University of California Riverside (UCR) time-series classification archive [79].

### Probabilistic finite automata

**Definition 1** (PFSA). *A probabilistic finite-state automaton G is a quadruple* $\left(Q,\Sigma ,\delta ,\tilde{\pi }\right)$, *where Q is a finite set of states*, Σ *is a finite alphabet, δ*: *Q* × Σ → *Q called transition map, and* $\tilde{\pi }:Q\times \Sigma \to \left[0,1\right]$ *specifies observation probabilities, with* $\forall q\in Q,\sum_{\sigma \in \Sigma }\tilde{\pi }\left(q,\sigma \right)=1$.

We use lower case Greeks (e.g. *σ* or *τ*) for symbols in Σ and lower case Latins (e.g. *x* or *y*) to denote sequence of symbols, with the empty sequence denoted by λ. The length of a sequence *x* is denoted by |*x*|. The set of sequences of length *d* is denoted by Σ^*d*^.

The directed graph (not necessarily simple with possible loops and multi-edges) with vertices in *Q* and edges specified by *δ* is called the graph of the PFSA and, unless stated otherwise, assumed to be strongly connected [80].

**Definition 2** (Observation and Transition Matrices). *Given a PFSA* $\left(Q,\Sigma ,\delta ,\tilde{\pi }\right)$, *the observation matrix* ${\tilde{\Pi }}_{G}$ *is the* |*Q*| × |Σ| *matrix with the* (*q*, *σ*)-*entry given by* $\tilde{\pi }\left(q,\sigma \right)$, *and the transition matrix* Π_*G*_ *is the* |*Q*| × |*Q*| *matrix with the* (*q*, *q*′)-*entry, written as π*(*q*, *q*′), *given by* $\pi (q,q^{\prime })=\sum_{\sigma :\delta (q,\sigma )=q^{\prime }}\tilde{\pi }(q,\sigma )$.

Both *Π*_*G*_ and ${\tilde{\Pi }}_{G}$ are stochastic, *i*.*e*. non-negative with rows of sum 1. Since the graph of a PFSA is strongly connected, there is a unique probability vector **p**_*G*_ that satisfies $p_{G}^{T}\Pi_{G}=p_{G}^{T}$ [81], and is called the stationary distribution of *G*.

**Definition 3** (Γ-Expression). *δ and* $\tilde{\pi }$ *may be encoded by a set of* |*Q*| × |*Q*| *matrices* **Γ** = {Γ_*σ*_|*σ* ∈ Σ}, *where*

$$\begin{array}{c} \Gamma_{\sigma }{|}_{q,q^{\prime }}=\{\begin{array}{cc} \tilde{\pi }\left(q,\sigma \right) & if\;\delta \left(q,\sigma \right)=q^{\prime },\\ 0 & if\;otherwise. \end{array} \end{array}$$ (6)

*We extend the definition of the* Γ *to* Σ^⋆^ *by* $\Gamma_{x}=\prod_{i=1}^{n}\Gamma_{\sigma_{i}}$ *for x* = *σ*_1_…*σ*_*n*_ *with* Γ_λ_ = *I*, *the identity matrix*.

**Definition 4** (Sequence-Induced Distributions). *For a PFSA* $G=(Q,\Sigma ,\delta ,\tilde{\pi })$, *the distribution on Q induced by a sequence x is given by* $p_{G}^{T}\left(x\right)=〚p_{G}^{T}\Gamma_{x}〛$, *where* 〚**v**〛 = **v**/‖**v**‖_1_.

**Definition 5** (Stochastic process Generated by PFSA). *Let* $G=(Q,\Sigma ,\delta ,\tilde{\pi })$ *be a PFSA, the* Σ-*valued stochastic process* {*X*_*t*_}_*t*∈Σ_ *generated by G satisfies that X*_1_ *follows the distribution* $p_{G}^{T}{\tilde{\Pi }}_{G}$ *and X*_*t*+1_ *follows the distribution* $p_{G}{\left(X_{1}\cdots X_{t}\right)}^{T}{\tilde{\Pi }}_{G}$ *for* t∈N.

We denote the probability an PFSA *G* producing a sequence *x* by *p*_*G*_(*x*). We can verify that $p_{G}\left(x\right)={∥p_{G}^{T}\Gamma_{x}∥}_{1}$.

### Learning PFSA from sample paths

A key step in our approach is the abductive inferrence of a PFSA [82] from quantized incidence time series. Importantly, we do not specify the number of states, or the transition structure of the model; both the transition map and the observation probabilities are inferred from the observed data streams. A single PFSA modeling the incidence dynamics in high risk counties is obtained in step 5) of the procedure outlined in the section “Calculation of UnIT Risk” below. Importantly, we have a data stream for each county inferred to have a high initiation risk (Step 3). Thus, we infer a single PFSA from multiple data streams, where each data stream is assumed to be a sample path generated by a similar underlying process. The inference algorithm cited above is designed to take advanatge of multiple data stream inputs to identify a common model.

### Sequence likelihood divergence

**Definition 6** (Entropy rate and KL divergence). *The entropy rate of a PFSA G is the entropy rate of the stochastic process G generates* [83]. *Similarly, the KL divergence of a PFSA G*′ *from the PFSA G is the KL divergence of the process generated by the G*′ *from that of G. More precisely, we have the*

$$\begin{array}{c} H\left(G\right)=-\lim_{d\to \infty }\frac{1}{d}\sum_{x\in \Sigma^{d}}p_{G}\left(x\right)\log p_{G}\left(x\right), \end{array}$$ (7)

*and the KL divergence*

$$\begin{array}{c} D(G\;∥\;G^{\prime })=\lim_{d\to \infty }\frac{1}{d}\sum_{x\in \Sigma^{d}}p_{G}\left(x\right)\log\frac{p_{G}\left(x\right)}{p_{G^{\prime }}\left(x\right)}, \end{array}$$ (8)

*whenever the limits exist*.

We also refer to the KL divergence between stochastic processes as the Sequence Likelihood divergence (SLD).

**Definition 7** (Log-likelihood). *The log-likelihood* [83] *of a PFSA G generating x* ∈ Σ^*d*^ *is given by*

$$\begin{array}{c} L\left(x,G\right)=-\frac{1}{d}\log p_{G}\left(x\right). \end{array}$$ (9)

Algorithm A in S1 Text outlines the steps in computing *L*(*x*, *G*). The time complexity of log-likelihood evaluation is *O*(|*x*| × |*Q*|) with input length *x* and |*Q*| being the size of the PFSA state set.

**Theorem 1** (Convergence of Log-likelihood). *Let G and G*′ *be two irreducible PFSA, and let x* ∈ Σ^*d*^ *be a sequence generated by G. Then we have*

$$\begin{array}{c} L\left(x,G^{\prime }\right)\to H\left(G\right)+D(G\;∥\;G^{\prime }), \end{array}$$

*in probability as d* → ∞.

*Proof*. See proof of convergence in S1 Text.

### From distance matrix to similarity matrix

Let *D* be the pair-wise distance matrix with *d*_*ij*_ = Θ(*s*_*i*_, *s*_*j*_), where *s*_*i*_ is the flu time series of county *c*_*i*_. Then the affinity matrix *A* for spectral clustering is chosen as $a_{ij}=\exp(-d_{ij}^{2}/2)$.

### Data source: COVID-19 incidence & putative factors

Data on confirmed cases of COVID-19 were compiled and released at the COVID-19 Data Repository by the Center for Systems Science and Engineering at Johns Hopkins University (https://github.com/CSSEGISandData/COVID-19). The John Hopkins COVID-19 data represent data collated by the US Centers for Disease Control & Prevention (CDC) from individual states and local health agencies. Using the John Hopkins COVID-19 data resource, we obtained county-level confirmed new weekly case counts for all weeks upto the current point in time (2021–05-30) for 3094 US counties. We calculated COVID-19 case per capita using the 2019 population estimate provided by the US Census Bureau generated from 2010 US decennial census (https://www.census.gov/data/datasets/time-series/demo/popest/2010s-counties-detail.html).

We include five demographic independent variables: 1) total population, 2) percent of the total population aged 65+, 3) percent of Hispanics in the total population, 4) percent of black/African-American in the total population, 5) percent of minority groups in the total population. For socioeconomic factors, we consider: 1) percent of the total population in poverty and 2) median household income, Which are also obtained from the US Census Bureau, based on the 2010 US decennial census.

### Data source: Seasonal influenza incidence

The source of incidence counts for seasonal flu epidemic is the Truven MarketScan database [55]. This US national database collating data contributed by over 150 insurance carriers and large, self-insuring companies, contains over 4.6 billion inpatient and outpatient service claims, with over six billion diagnostic codes. We processed the Truven database to obtain the reported weekly number of influenza cases over a period of 471 weeks spanning from January 2003 to December 2011, at the spatial resolution of US counties. Standard ICD9 diagnostic codes corresponding to Influenza infection is used to determine the county-specific incidence time series, which are: 1) **487** Influenza, 2) **487.0** Influenza with pneumonia, and 3) **487.1** Influenza with other respiratory manifestations and 4) **487.8** Influenza with other manifestations.

### Discretization of incidence counts

Integer-valued incidence input is quantized to produce data streams with a finite alphabet, by choosing *k* − 1 cut-off points *p*_1_ < *p*_2_ < ⋯ < *p*_*k*−1_ and replacing a value <*p*_1_ by 0, in [*p*_*i*_, *p*_*i*+1_) by *i*, and ≥ *p*_*k*−1_ by *k*. We call the set of cut-off points a *partition*. In our processing of incidence count data for flu epidemics, we obtain a binary partition by first taking a 1-step difference (*i*.*e*., transforming a length-*n* sequence *x*_1_, *x*_2_, …, *x*_*n*−1_, *x*_*n*_ to *x*_2_ − *x*_1_, *x*_3_ − *x*_2_, …, *x*_*n*_ − *x*_*n*−1_), and then replacing each positive value in the resulting sequence by 1 and the remaining, 0. Thus weeks with a rise in count is marked by 1, and teh remianing by 0.

### Calculation of UnIT risk

We estimate the UnIT risk via the following 6 steps: 1) Compute pairwise similarity between US counties using the metric Θ introduced in Eq (5). 2) Cluster counties using this similarity measure using standard spectral clustering algorithm [56]. 3) Identify the set of counties that have high initiation risk, defined as ones that report cases within the first two weeks of each flu season. 4) Identify the cluster that has a maximal overlap with the set of high-risk counties. If we infer 4 clusters, then we found that only one cluster is sufficient to represent the set of high risk counties. If we set the parameters of the clustering algorithm to find more clusters, then more than one “high-risk” cluster might emerge, which we then collapse and treat as a single set for the next steps. 5) Generate a single PFSA *G*^⋆^ based on the quantized incidence series from counties in the high-risk cluster cluster, using a reported abductive inference algorithm [54]. 6) Finally, estimate UnIT risk as

$$\begin{array}{c} \hat{\nu (x)}≜L\left(x,G^{⋆}\right)-\hat{H(X)}\to D(X∥G^{⋆}) \end{array}$$ (10)

The entropy rate is estimated as the entropy of the distribution of 0s and 1s (length 2 probability vector enumerating the fraction of 0s vs 1s), which provides an upper bound to the entropy rate [53]. Thus, our estimate for the UnIT risk gives us a lower bound, and more detailed computation only improves results marginally.

### Calculation of urban-UnIT risk

In our modeling and forecasting investigations pertaining to the problem at hand, we use a scaled version of the UnIT risk denoted as the urban-UnIT risk, which is the county-wise product of the UnIT risk with the fraction of the population living in urban environment, as estimated from the 2010 US census.

### UnIT correction to case count forecast

We fit a generalized linear model [40, 84] (GLM) with the assumption that the response variable (county specific weekly case counts confirmed for COVID-19) follows a Poisson distribution, and that the logarithm of its expected value can be modeled by a linear combination of unknown parameters.

Specifically, if the response *Y*, is assumed to be a count that follows a Poisson distribution with mean *μ*, then:

$$\begin{array}{c} \text{log}\left(\mu \right)=\beta_{0}+\beta_{1}X_{1}+\beta_{2}X_{2}+\cdots +\beta_{k}X_{k}, \end{array}$$ (11)

where *X*_1_, *X*_2_, …, *X*_*k*_ are explanatory variables (covariates). The counts are for all one-week periods between 2020-04-04 to 2021-05-30. This is also known as Poisson regression or a log-linear model.

To investigate the predictive contribution of the UnIT risk, we explore two models: 1) *Baseline model* with the following demographic and socio-economic covariates: percentage of urban population, population, percentage of population above 65 years old, percentage of minority population, percentage of black population, percentage of Hispanic population, percentage of population in poverty, median household income; and 2) *UnIT-Augmented model* which includes the covariates in the baseline model, with the additional urban-UnIT risk factor discussed above. Note that for the GLM modeling, we use standard score for all covariates and dependent variables with zero mean and unit variance, *i*.*e*., assuming the data for a variable is *x*_1_, …, *x*_*n*_ and let $\hat{\mu }$ and $\hat{\sigma }$ be the sample mean and sample standard deviation, respectively, we transform *x*_*i*_ to $\left(x_{i}-\hat{\mu }\right)/\hat{\sigma }$, so that a comparison of the magnitudes of the coefficients reflect the relative importance of the significant covariates.

As described before, we use the GLM model to obtain a “corrected” version of the county-specific case count vector, which is subsequently used to train an ensemble regressor to predict case counts 1 week into future. The precise algorithmic steps are enumerated in Algorithm B in S1 Text. To reduce variance we train a set R of regressors in the final step, and report the mean. Here R consists of a random forest model, an extra trees model and a feed-forward neural network model with a single hidden layer implemented through Tensor Flow.

### Forecasting COVID-19-related deaths

An almost identical approach is used to forecast COVID-19-related deaths, where we use the same covariates as before, but replace the county-specific case count vector with the county-specific record of COVID-19-related deaths. The modified algorithm for forecasting deaths is enumerated in Algorithm C in S1 Text, where for training regressors, we also use the case count vectors and its corrected version produced by Algorithm B in S1 Text.

## Supporting information

S1 Fig Additional time-points for comparing the ranking of teams on the COVID-19 forecasthub via mean absolute forecast errors measured over 1-week forecasts from the start of the pandemic (or when the specific team starts reporting). UnIT score dominates, except at the early months of the pandemic. Additionally, the approach with Poisson regression at the first stage dominates over using negative binomial regression, despite indications that the data is somewhat overdispersed. **Panel B** shows the % forecast error achieved over time, along with a LOWESS fit. Note that we can see three distinct zones: upto mid-June in 2020 we have a slight under-estimation, and after we reached peak infection in the US (*i*.*e*. after ≈ Jan 5 2021), we see a slight over-estimation of the case counts, with the average estimation errors close to zero in the intervening period. This variation might reflect varying effectiveness of the UnIT risk over the pandemic timeline. However, applying our approach to reported “nowcast” estimates that correct for reporting errors, under-testing and other factors that obfuscate the case count, we find that these trends disappear (S2 Fig), suggesting reporting inaccuracies to be a significant contributor to these trends.
(TIF)

S2 Fig **Panel A**. Applying the methodology in this paper to forecasting weekly HIV cases as a test of generalizability. Prediction errors are relatively large: the mean absolute error as a fraction of the number of weekly case can be as high as 63.12% (11.9% on average), whereas in the case of COVID-19 prediction this is limited to 23.8% (9.5% on average). While we can track the trend well on average, this is of less practical value compared to the scenario of a rapidly spreading acute infection such as COVID-19. The worse performance here stems from the differences in infection mechanisms of influenza and HIV, and also perhaps the epidemiology of HIV which presents as a chronic infection, with potentially longer time to serroconversion (< 2 months [64]), making weekly predictions not particularly appropriate. **Panel B** illustrates that the COVID-19 case prediction works equally well if we use nowcast estimates (as reported at https://covid19-projections.com/infections/summary-counties/) as the ground truth, instead of case reports curated at the covid forecasthub. **Panel C** illustrates that the % forecast errors are significantly trend-free, with the LOWESS fit staying close to zero.
(TIF)

S3 Fig To test the robustness of the UnIT score as a key influencing variable, we tested two perturbation modes. (left column) randomly selecting only 75% of the counties to include in the analysis (considered along with 99% confidence bounds), and (right column) deleting the top 10% of the counties ranked by the highest number of COVID-19 cases per capita. As shown in panels A and B, under all such perturbations, the UnIT score retains its position as the dominant factor in our regression models, measured by the magnitude of the inferred coefficient relative to those of the other covariates. In particular, in panel A, subpanels (i) and (ii) show the variation of the coefficients for the baseline model for the two perturbation modes described above. The covariates considered in the baseline models are those enumerated in Table 1 in the main text with the exception of the UnIT risk variables. The corresponding plots for theUnIT-augmented model which includes the additional UnIT risk and urban-UnIT risk as covariates is shown in subpanels (iii) and (iv). Panel B shows the explained variation in the models for the two perturbation modes in panels and panel C illustrates the outperformance in explained variance.
(TIF)

S4 Fig **Panel A**. Forecast accuracy of COVID-19-related confirmed deaths measured by mean absolute error of top-performing teams in the COVID-19 forecasthub. **Panel B**. Death count forecasts made by our model against the ground truth. The somewhat reduced effectiveness of our death forecast is probably attributable to the differences between the clinical progression of Influenza and COVID-19.
(TIF)

S5 Fig**Panel A-D** Four pre-specified PFSAs to estimate similarity between stochastic sample paths (See Eq (5) in main text). An edge connecting state *q* to *q*′ is labeled as $\sigma (\tilde{\pi }\left(q,\sigma \right))$ if *δ*(*q*, *σ*) = *q*′ (See Defn. 1). **Panel e**. Performance and run time comparisons of SLD distance and DTW on a synthetic dataset. We denote the SLD distance by the length of the input sequence and DTW by their window size in Panel e. The average run time of of SLD distance is.042 second. **Panel f**. Run time v.s. sequence length comparison between DTW30 and the SLD distance. Panel g: 2*D* embeddings produced by Algorithm A in S1 Text and DTW5 on the “FordA” dataset from the UCR time series classification archive [79] with decision boundaries obtained by using Support Vector Machines (SVM) and neural networks respectively trained with features constructed from the corresponding dissimilarity measures. The SLD approach yields significantly improved separation.(TIF)

S1 Text Text with supplementary tables, pseudocode, sofware usage instructions, and proof of Theorem 1. **Table A**: COVID-19 ForecastHub (https://covid19forecasthub.org/community) Community Team Summary. **Table B**: Coefficients in multi-variate regression for COVID-19-related death count total as of 2021–05-30. **Table C**: Coefficients inferred in multi-variate regression for weekly COVID-19-related death totals. List of Algorithm Pseudocodes. **Algorithm A**: PFSA Log-likelihood. **Algorithm B**: Weekly confirmed case forecasting. **Algorithm C**: Weekly death forecasting.
(PDF)
