# Supplementary figures and images for: Universal risk phenotype of US counties for flu-like transmission to improve county-specific COVID-19 incidence forecasts
Source: PLoS Comput Biol. 2021 Oct 14;17(10):e1009363. doi: 10.1371/journal.pcbi.1009363 (PMC8516313; doi:10.1371/journal.pcbi.1009363)

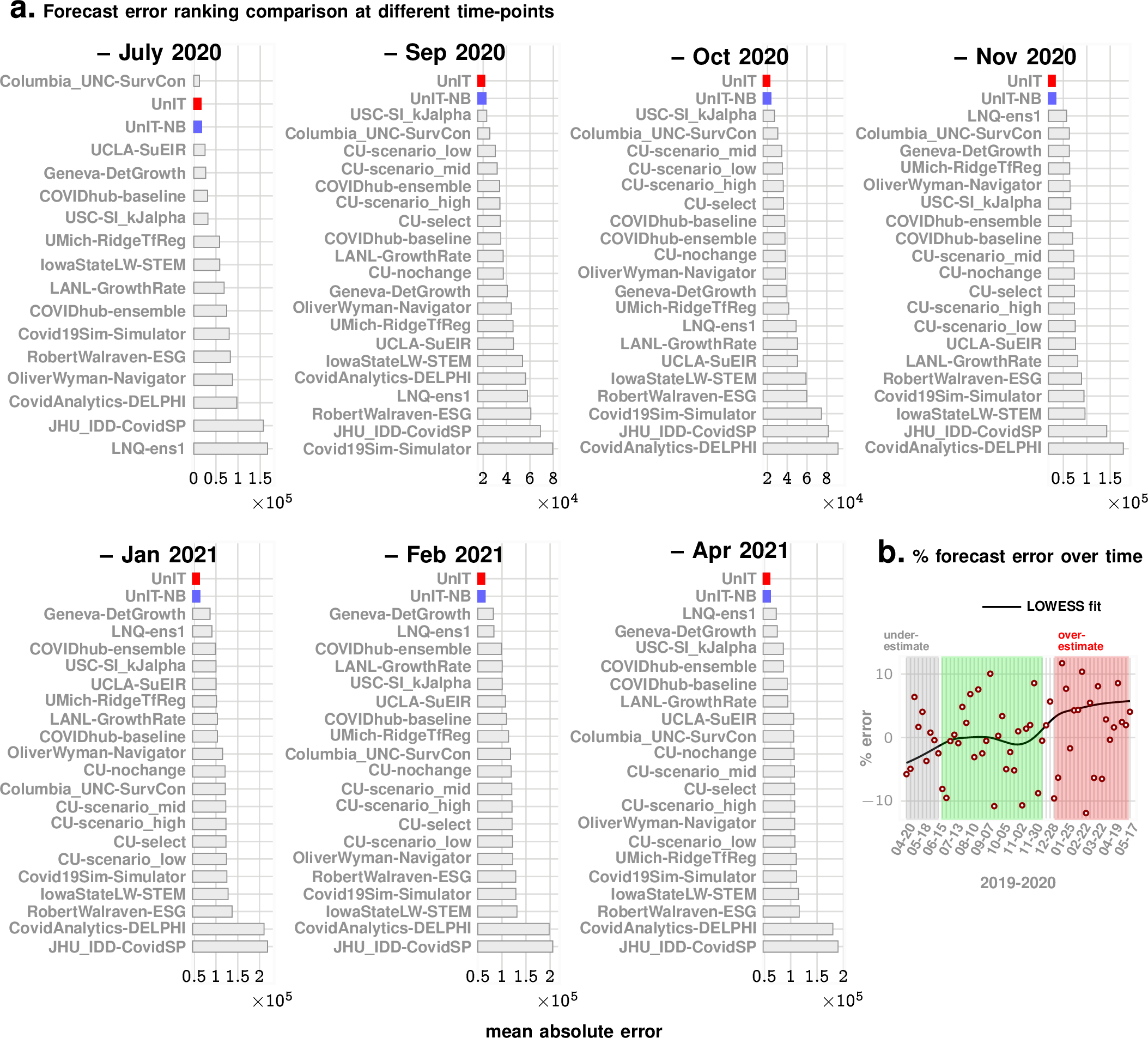

Supplement: S1 Fig — UnIT score dominates, except at the early months of the pandemic. Additionally, the approach with Poisson regression at the first stage dominates over using negative binomial regression, despite indications that the data is somewhat overdispersed. Panel B shows the % forecast error achieved over time, along with a LOWESS fit. Note that we can see three distinct zones: upto mid-June in 2020 we have a slight under-estimation, and after we reached peak infection in the US (i.e. after ≈ Jan 5 2021), we see a slight over-estimation of the case counts, with the average estimation errors close to zero in the intervening period. This variation might reflect varying effectiveness of the UnIT risk over the pandemic timeline. However, applying our approach to reported “nowcast” estimates that correct for reporting errors, under-testing and other factors that obfuscate the case count, we find that these trends disappear (S2 Fig), suggesting reporting inaccuracies to be a significant contributor to these trends. (TIF) [file pcbi.1009363.s001.tif]

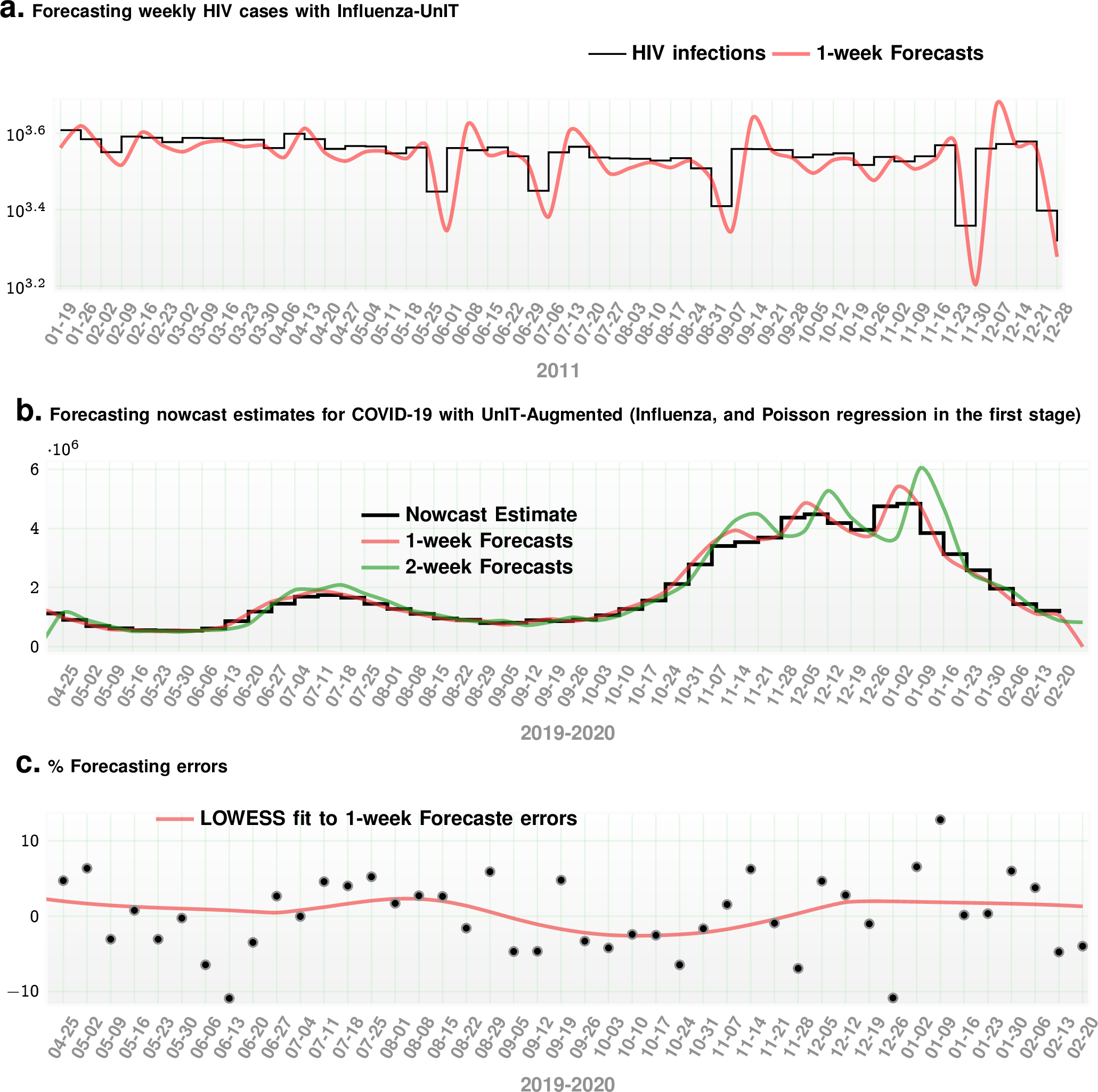

Supplement: S2 Fig — Panel A. Applying the methodology in this paper to forecasting weekly HIV cases as a test of generalizability. Prediction errors are relatively large: the mean absolute error as a fraction of the number of weekly case can be as high as 63.12% (11.9% on average), whereas in the case of COVID-19 prediction this is limited to 23.8% (9.5% on average). While we can track the trend well on average, this is of less practical value compared to the scenario of a rapidly spreading acute infection such as COVID-19. The worse performance here stems from the differences in infection mechanisms of influenza and HIV, and also perhaps the epidemiology of HIV which presents as a chronic infection, with potentially longer time to serroconversion (< 2 months [64]), making weekly predictions not particularly appropriate. Panel B illustrates that the COVID-19 case prediction works equally well if we use nowcast estimates (as reported at https://covid19-projections.com/infections/summary-counties/) as the ground truth, instead of case reports curated at the covid forecasthub. Panel C illustrates that the % forecast errors are significantly trend-free, with the LOWESS fit staying close to zero. (TIF) [file pcbi.1009363.s002.tif]

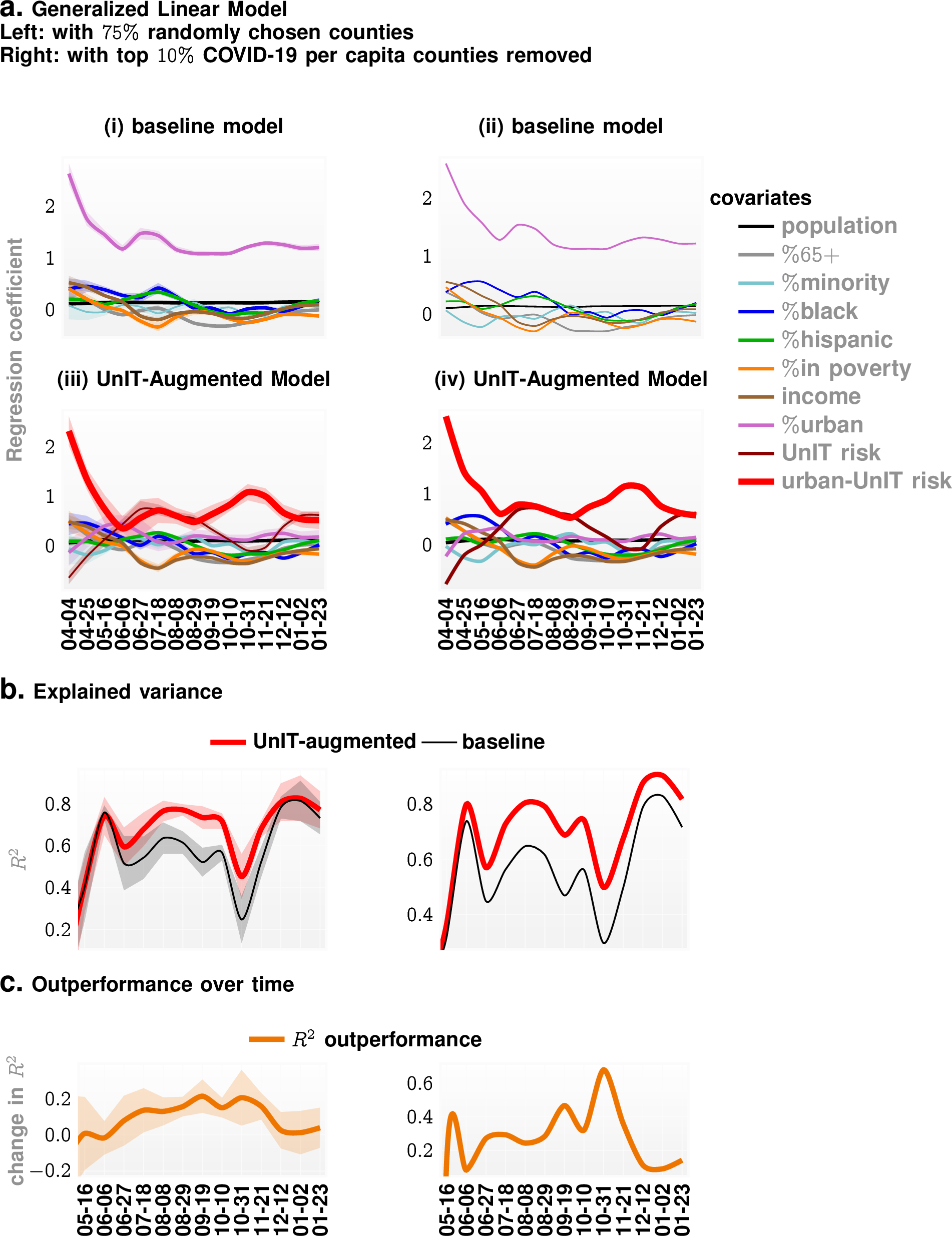

Supplement: S3 Fig — (left column) randomly selecting only 75% of the counties to include in the analysis (considered along with 99% confidence bounds), and (right column) deleting the top 10% of the counties ranked by the highest number of COVID-19 cases per capita. As shown in panels A and B, under all such perturbations, the UnIT score retains its position as the dominant factor in our regression models, measured by the magnitude of the inferred coefficient relative to those of the other covariates. In particular, in panel A, subpanels (i) and (ii) show the variation of the coefficients for the baseline model for the two perturbation modes described above. The covariates considered in the baseline models are those enumerated in Table 1 in the main text with the exception of the UnIT risk variables. The corresponding plots for theUnIT-augmented model which includes the additional UnIT risk and urban-UnIT risk as covariates is shown in subpanels (iii) and (iv). Panel B shows the explained variation in the models for the two perturbation modes in panels and panel C illustrates the outperformance in explained variance. (TIF) [file pcbi.1009363.s003.tif]

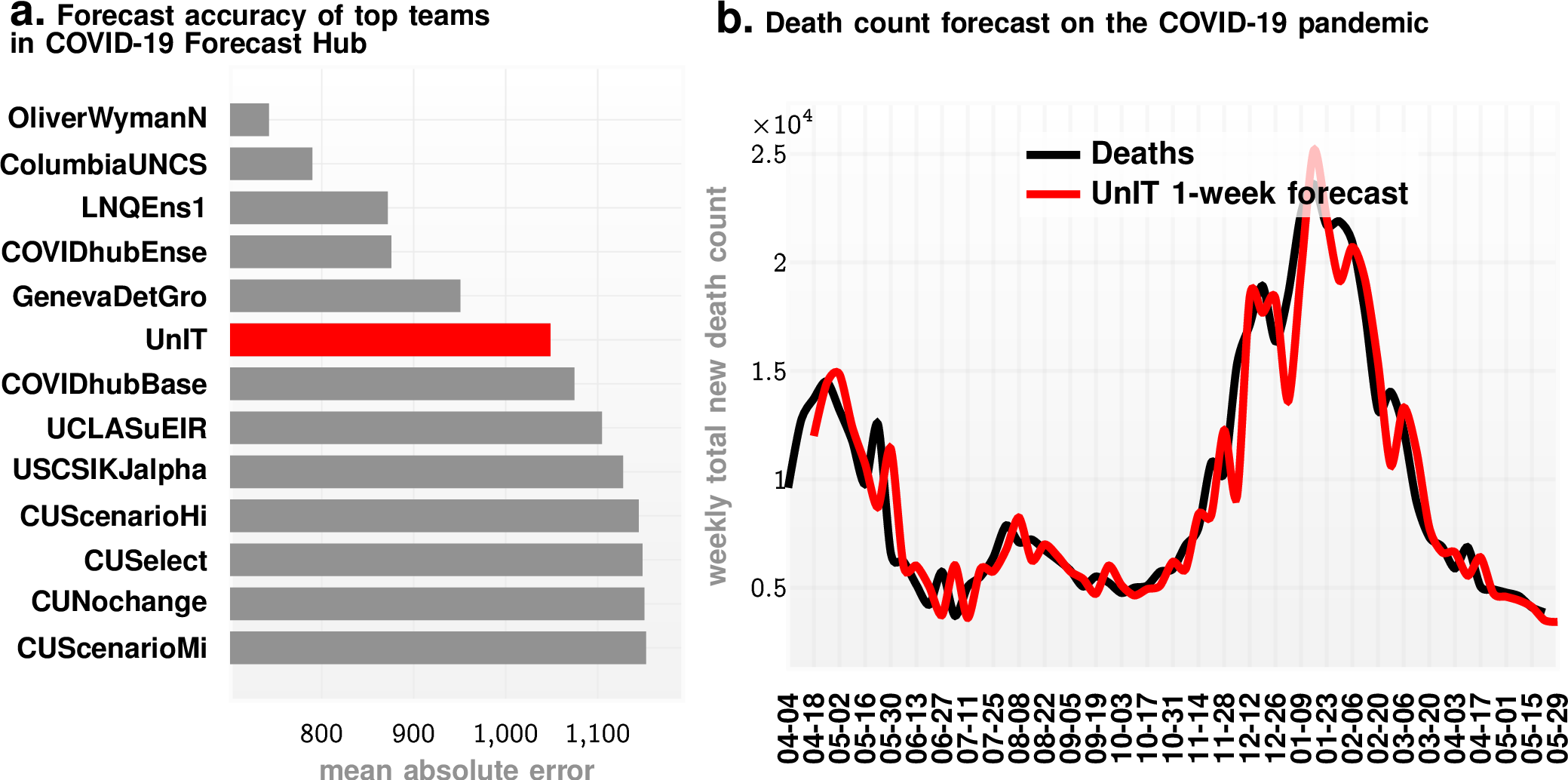

Supplement: S4 Fig — Panel A. Forecast accuracy of COVID-19-related confirmed deaths measured by mean absolute error of top-performing teams in the COVID-19 forecasthub. Panel B. Death count forecasts made by our model against the ground truth. The somewhat reduced effectiveness of our death forecast is probably attributable to the differences between the clinical progression of Influenza and COVID-19. (TIF) [file pcbi.1009363.s004.tif]

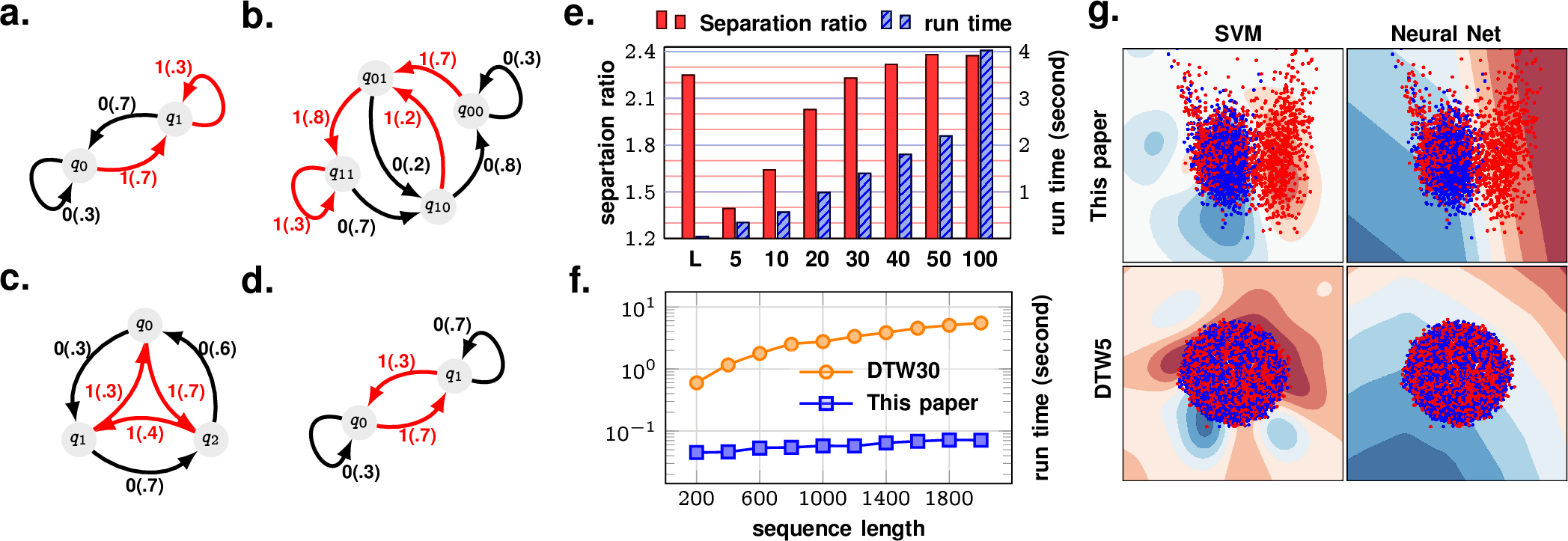

Supplement: S5 Fig — Panel A-D Four pre-specified PFSAs to estimate similarity between stochastic sample paths (See Eq (5) in main text). An edge connecting state q to q′ is labeled as σ(π˜(q,σ)) if δ(q, σ) = q′ (See Defn. 1). Panel e. Performance and run time comparisons of SLD distance and DTW on a synthetic dataset. We denote the SLD distance by the length of the input sequence and DTW by their window size in Panel e. The average run time of of SLD distance is.042 second. Panel f. Run time v.s. sequence length comparison between DTW30 and the SLD distance. Panel g: 2D embeddings produced by Algorithm A in S1 Text and DTW5 on the “FordA” dataset from the UCR time series classification archive [79] with decision boundaries obtained by using Support Vector Machines (SVM) and neural networks respectively trained with features constructed from the corresponding dissimilarity measures. The SLD approach yields significantly improved separation. (TIF) [file pcbi.1009363.s005.tif]
