# Supplementary material for: Universal risk phenotype of US counties for flu-like transmission to improve county-specific COVID-19 incidence forecasts
Source: PLoS Comput Biol. 2021 Oct 14;17(10):e1009363. doi: 10.1371/journal.pcbi.1009363 (PMC8516313; doi:10.1371/journal.pcbi.1009363)
Supplement: S1 Text — Table A: COVID-19 ForecastHub (https://covid19forecasthub.org/community) Community Team Summary. Table B: Coefficients in multi-variate regression for COVID-19-related death count total as of 2021–05-30. Table C: Coefficients inferred in multi-variate regression for weekly COVID-19-related death totals. List of Algorithm Pseudocodes. Algorithm A: PFSA Log-likelihood. Algorithm B: Weekly confirmed case forecasting. Algorithm C: Weekly death forecasting. (PDF) [file pcbi.1009363.s006.pdf]

Algorithm A: PFSA Log-likelihood

Data: A PFSA $G = (Q, \Sigma, \delta, \widetilde{\pi})$ and a sequence x of length d.
Result: Log-likelihood of G generating x

```
Get the stationary distribution p_G as the left eigenvector of Π_G of eigenvalue 1;
Let p be the current distribution on states, and initialize it with p_G;
Let L be the log-likelihood of G generating x and initialize it with 0;
for each symbol σ in x do
    Get the current distribution on symbols φ = p_G^T Π̃_G;
    Update L = L − log φ(σ);
    Let p_new be the new distribution on states, and initialize all its entries with 0;
    for each state q ∈ Q do
        Let the next the state q_new = δ(q, σ);
        Let p_new(q_new) = p_new(q_new) + p(q) π̃(q, σ);
    Update p with p_new / ||p_new||_1;
Let L = L/d;
return L;
```

Algorithm B: Weekly confirmed case forecasting

Data:
- X_{t-1}: vector of confirmed county-wise cases at time point $t-1$;
- X_t: vector of confirmed county-wise cases at time point t;
- $X^{\star}_{t-1}$ and $X^{\star}_t$: predicted vector of county-wise cases from by the GLM model for time points $t-1$ and t;
- A set $\mathcal{R}$ of regressor functions (we choose a random forest, and an extremely randomized trees model);

Result: $\widehat{X}_{t+1}$, forecast of confirmed cases in time period $t+1$ for each county.

for *each regressor* $\texttt{Regr} \in \mathcal{R}$ **do**
  Let $X_{\text{train}} = \left[X_{t-1}, X^{\star}_{t-2}, X^{\star}_{t-1}\right]$;
  Let $y_{\text{train}} = X_t$;
  Train $\texttt{Regr}$ with $X_{\text{train}}, y_{\text{train}}$;
  $y_{\text{pred},\texttt{Regr}} = \texttt{Regr}\left(\left[X_t, X^{\star}_{t-1}, X^{\star}_t\right]\right)$;
return $\widehat{X}_{t+1} = \sum_{r \in \mathcal{R}} y_{\text{pred},r} / |\mathcal{R}|$;

Algorithm C: Weekly death forecasting

Data:
- D_{t-1} and D_t: vector of county-wise deaths at $t-1$ and t;
- X_{t-1} and X_t: vector of confirmed county-wise cases at $t-1$ and t;
- $D^{\star}_{t-1}$ and $D^{\star}_t$: predicted county-wise deaths given by GLM model at $t-1$ and t;
- $X^{\star}_{t-1}$ and $X^{\star}_t$: predicted county-wise cases by GLM model at $t-1$ and t;
- A set $\mathcal{R}$ of regressors (we choose a random forest, and an extremely randomized trees model);

Result: $\widehat{D}_{t+1}$, forecast of death in time period $t+1$ for each county.

for *each regressor* $\texttt{Regr} \in \mathcal{R}$ **do**
  Let $X_{\text{train}} = \left[D_{t-1}, D^{\star}_{t-2}, D^{\star}_{t-1}, X_{t-1}, X^{\star}_{t-2}, X^{\star}_{t-1}\right]$;
  Let $y_{\text{train}} = D_t$;
  Train $\texttt{Regr}$ with $X_{\text{train}}, y_{\text{train}}$;
  $y_{\text{pred},\texttt{Regr}} = \texttt{Regr}\left(\left[D_t, X_t, D^{\star}_{t-1}, D^{\star}_t, X^{\star}_{t-1}, X^{\star}_t\right]\right)$;
return $\widehat{D}_{t+1} = \sum_{r \in \mathcal{R}} y_{\text{pred},r} / |\mathcal{R}|$;

SOFTWARE USAGE INSTRUCTIONS

The complete software is available at https://github.com/zeroknowledgediscovery/unitcov. The following steps are required to download, install and execute our model (on a Linux OS with python 3 and jupyter notebook installation) to obtain the next week's case count and COVID-19-related death count estimates:

1) `git clone https://github.com/zeroknowledgediscovery/unitcov.git`
2) `cd unitcov/forecast_pipeline`
3) `jupyter notebook`

Then, in the jupyter notebook environment, execute the following:

1) `pipeline_data_gathering.ipynb`
2) `pipeline_GLM.ipynb`
3) `pipeline_forecast_case.ipynb` and `pipeline_forecast_death.ipynb`

Alternatively, one can run `forecast_for_next_week.ipynb` which is a combination of the steps above.

PROOF OF CONVERGENCE

Theorem A (Proof of Convergence of Log-likelihood). *Let G and G' be two irreducible PFSA, and let $x \in \Sigma^d$ be a sequence generated by G. Then we have*

$$L(x, G') \to G'(G) + \mathcal{D}(G \,||\, G'),$$

in probability as $d \to \infty$.

Proof: By chain rule

$$\begin{aligned}
&\sum_{x \in \Sigma^d} p_G(x) \log \frac{p_G(x)}{p_{G'}(x)} \\
&= \sum_{x \in \Sigma^{d-1}} \sum_{\sigma \in \Sigma} p_G(x) \mathbf{p}_G^T(x) \left.\widetilde{\Pi}_G\right|_\sigma \log \frac{p_G(x) \mathbf{p}_G(x)^T \left.\widetilde{\Pi}_G\right|_\sigma}{p_{G'}(x) \mathbf{p}_{G'}(x)^T \left.\widetilde{\Pi}_{G'}\right|_\sigma} \\
&= \sum_{x \in \Sigma^{d-1}} p_G(x) \log \frac{p_G(x)}{p_{G'}(x)} \\
&\quad + \underbrace{\sum_{x \in \Sigma^{d-1}} p_G(x) \sum_{\sigma \in \Sigma} \mathbf{p}_G(x)^T \left.\widetilde{\Pi}_G\right|_\sigma \log \frac{\mathbf{p}_G(x)^T \left.\widetilde{\Pi}_G\right|_\sigma}{\mathbf{p}_{G'}(x)^T \left.\widetilde{\Pi}_{G'}\right|_\sigma}}_{D_d}.
\end{aligned}$$

By induction, we have $\mathcal{D}(G \,||\, G') = \lim_{d\to\infty} \frac{1}{d} \sum_{i=1}^d D_i$, and hence by Cesàro summation theorem (8), we have

$$\mathcal{D}(G \,||\, G') = \lim_{d \to \infty} D_d.$$

If $x = \sigma_1 \sigma_2 ... \sigma_n$ is generated by G and $x^{[i-1]}$ is the truncation of x at the $(i-1)$-th symbol, we have

$$-\frac{1}{n} \sum_{i=1}^n \log \mathbf{p}_{G'} \left(x^{[i-1]}\right)^T \left.\widetilde{\Pi}_{G'}\right|_{\sigma_i} = \underbrace{\frac{1}{n} \sum_{i=1}^n \log \frac{\mathbf{p}_G \left(x^{[i-1]}\right)^T \left.\widetilde{\Pi}_G\right|_{\sigma_i}}{\mathbf{p}_{G'} \left(x^{[i-1]}\right)^T \left.\widetilde{\Pi}_{G'}\right|_{\sigma_i}}}_{A_{x,n}} - \underbrace{\frac{1}{n} \sum_{i=1}^n \log \mathbf{p}_G \left(x^{[i-1]}\right)^T \left.\widetilde{\Pi}_G\right|_{\sigma_i}}_{B_{x,n}}.$$

Because the process generated by G is ergodic, we have

$$\lim_{n \to \infty} A_{x,n} = \lim_{d \to \infty} D_d = \mathcal{D}(G \,||\, G'). \tag{1}$$

and $\lim_{n\to\infty} B_{x,n} = H(G)$. ∎

TABLE A
COEFFICIENTS IN MULTI-VARIATE REGRESSION FOR COVID-19-RELATED DEATH COUNT TOTAL AS OF 2021-05-30

	coef.	z-value	.025	.975
pop	0.083	319.679	0.082	0.083
%65+	0.185	104.744	0.182	0.189
%minority	0.146	42.327	0.140	0.153
%black	−0.072	−22.048	−0.078	−0.065
%hispanic	0.040	28.342	0.037	0.043
%poverty	0.041	13.197	0.035	0.047
income	−0.067	−31.026	−0.071	−0.063
%urban	0.058	10.618	0.047	0.068
UnIT	0.401	78.380	0.391	0.411
urban UnIT	0.836	127.154	0.823	0.848

All p-values are < 0.0005.

TABLE B
COVID-19 FORECASTHUB (HTTPS://COVID19FORECASTHUB.ORG/COMMUNITY) COMMUNITY TEAM SUMMARY

Mean absolute error	Team name	Description	Link
230535	Johns Hopkins ID Dynamics COVID-19 Working Group (Abbr. JHU_IDD-CovidSP)	County-level metapopulation model with commuting and stochastic SEIR disease dynamics with social-distancing indicators.	https://github.com/HopkinsIDD/COVIDScenarioPipeline
197888	CovidAnalytics at MIT (Abbr. CovidAnalytics-DELPHI)	This model predicts based on an SEIR model augmented with underdetection and interventions. Projections account for reopening and assume interventions would be re-enacted if cases continue to climb.	https://www.covidanalytics.io/
124743	Robert Walraven (Abbr. RobertWalraven-ESG)	Multiple skewed gaussian distribution peaks fit to raw data	http://rwalraven.com/COVID19
116034	Columbia_UNC (Abbr. Columbia_UNC-SurvCon)	A survival-convolution model with piece-wise transmission rates that incorporates latent incubation period and provides time-varying effective reproductive number.	https://github.com/COVID19BIOSTAT/covid19_prediction
113322	Columbia University (Abbr. CU-select)	A metapopulation county-level SEIR model for projecting future COVID-19 incidence and deaths.	https://blogs.cuit.columbia.edu/jls106/publications/covid-19-findings-simulations/
110366	Los Alamos National Labs (Abbr. LANL-GrowthRate)	This model makes predictions about the future, unconditional on particular intervention strategies. Statistical dynamical growth model accounting for population susceptibility.	https://covid-19.bsvgateway.org/
109941	Iowa State - Lily Wang's Research Group (Abbr. IowaStateLW-STEM)	Spatiotemporal Dynamics, Nowcasting and Forecasting of COVID-19 in the United States.	https://covid19.stat.iastate.edu
107403	COVID-19 Simulator (Abbr. Covid19Sim-Simulator)	An interactive tool developed by researchers at Mass General Hospital, Harvard Medical School, Georgia Tech and Boston Medical Center to inform COVID-19 intervention policy decisions in the US.	https://covid19sim.org/
98801	UCLA Statistical Machine Learning Lab (Abbr. UCLA-SuEIR)	The SuEIR model is a variant of the SEIR model considering both untested and unreported cases. The model considers reopening and assumes susceptible population will increase after the reopen.	https://covid19.uclaml.org/
98472	University of Southern California Data Science Lab (Abbr. USC-SI_kJalpha)	A heterogeneous infection rate model with human mobility for epidemic modeling. Our model adapts to changing trends and provide predictions of confirmed cases and deaths.	https://scc-usc.github.io/ReCOVER-COVID-19
91689	The University of Michigan (Abbr. UMich-RidgeTfReg)	Nation-level model of confirmed cases and deaths based on ridge regression. No assumptions made about social distancing.	https://gitlab.com/sabcorse/covid-19-collaboration
91105	COVID-19 Forecast Hub (Abbr. COVIDhub-baseline)	This model is a baseline predictive model.	https://covid19forecasthub.org/
90969	Oliver Wyman (Abbr. OliverWyman-Navigator)	Oliver Wyman's Pandemic Navigator provides forecasts and scenario analysis for Detected and Undetected cases and death counts following a compartmental formulation with non-stationary transition rates.	https://pandemicnavigator.oliverwyman.com/
89285	LockNQuay (Abbr. LNQ-ens1)	County-level ensemble of boosted tree and neural net models. Lots of engineered features.	https://www.kaggle.com/sasrdw/locknquay
89197	University of Geneva / Swiss Data Science Center (Abbr. Geneva-DetGrowth)	Growth rate of cumulative cases (resp. deaths) between two days ago and today is estimated. If greater than 5%, use an exponential model to forecast. Otherwise, use a linear model.	https://renkulab.shinyapps.io/COVID-19-Epidemic-Forecasting/
87192	QJHong (Abbr. QJHong-Encounter)	today's Daily New Confirmed Cases + today's Encounter Density = today's newly infected Cases = next 2-3 weeks' Daily New Confirmed Cases	https://qjhong.github.io
85103	COVIDhub-ensemble	The ensemble prediction at COVID forecasthub	

TABLE C
COEFFICIENTS INFERRED IN MULTI-VARIATE REGRESSION FOR WEEKLY COVID-19-RELATED DEATH TOTALS

		pop	%65+	%minority	%black	%hispanic	%poverty	income	%urban	pre-UnIT	UnIT
10-10	z-value	22.4	2.84	−0.052	−1.21	4.71	−0.400	−9.53	1.48	10.1	9.61
	.025	0.067	0.017	−0.083	−0.122	0.039	−0.077	−0.307	−0.025	0.391	0.496
	.975	0.080	0.095	0.079	0.029	0.093	0.051	−0.202	0.183	0.581	0.750
	coef.	0.073	0.056	−0.002	−0.047	0.066	−0.013	−0.255	0.079	0.486	0.623
10-17	z-value	20.9	8.68	1.28	−1.47	5.26	−6.65	−13.7	−1.59	7.62	14.1
	.025	0.064	0.121	−0.029	−0.134	0.047	−0.299	−0.436	−0.174	0.253	0.734
	.975	0.077	0.191	0.136	0.019	0.104	−0.163	−0.327	0.018	0.428	0.970
	coef.	0.070	0.156	0.054	−0.057	0.075	−0.231	−0.381	−0.078	0.340	0.852
10-24	z-value	25.4	3.54	4.45	−5.75	−3.06	−8.02	−15.1	5.30	9.85	8.33
	.025	0.077	0.028	0.086	−0.252	−0.073	−0.329	−0.445	0.155	0.335	0.371
	.975	0.090	0.097	0.222	−0.124	−0.016	−0.200	−0.343	0.337	0.502	0.600
	coef.	0.084	0.062	0.154	−0.188	−0.045	−0.264	−0.394	0.246	0.419	0.486
10-31	z-value	22.3	−3.24	2.87	−8.23	−6.90	−7.26	−15.8	2.20	8.65	11.3
	.025	0.070	−0.098	0.032	−0.336	−0.129	−0.301	−0.465	0.011	0.278	0.535
	.975	0.084	−0.024	0.167	−0.207	−0.072	−0.173	−0.363	0.190	0.441	0.760
	coef.	0.077	−0.061	0.099	−0.272	−0.101	−0.237	−0.414	0.101	0.360	0.648
11-07	z-value	27.8	−6.11	−0.934	−2.69	−7.22	−3.86	−13.8	1.60	8.85	11.8
	.025	0.080	−0.145	−0.099	−0.148	−0.121	−0.166	−0.372	−0.015	0.258	0.509
	.975	0.092	−0.075	0.035	−0.023	−0.069	−0.054	−0.279	0.148	0.405	0.712
	coef.	0.086	−0.110	−0.032	−0.086	−0.095	−0.110	−0.326	0.067	0.331	0.610
11-14	z-value	26.9	−5.21	−3.20	−2.08	−7.42	−8.65	−16.7	1.41	6.29	15.7
	.025	0.075	−0.120	−0.189	−0.138	−0.119	−0.306	−0.428	−0.022	0.158	0.685
	.975	0.087	−0.054	−0.045	−0.004	−0.069	−0.193	−0.338	0.133	0.300	0.880
	coef.	0.081	−0.087	−0.117	−0.071	−0.094	−0.250	−0.383	0.056	0.229	0.782
11-21	z-value	34.0	−4.50	−5.23	−1.35	−9.82	−8.87	−18.5	5.17	4.34	18.5
	.025	0.080	−0.090	−0.231	−0.099	−0.128	−0.266	−0.398	0.107	0.075	0.711
	.975	0.090	−0.035	−0.105	0.018	−0.085	−0.170	−0.322	0.239	0.200	0.880
	coef.	0.085	−0.063	−0.168	−0.041	−0.106	−0.218	−0.360	0.173	0.138	0.795
11-28	z-value	35.7	−1.65	−3.23	−4.61	−10.3	−10.1	−18.8	3.42	3.90	19.8
	.025	0.083	−0.050	−0.165	−0.197	−0.139	−0.312	−0.417	0.049	0.063	0.778
	.975	0.093	0.004	−0.040	−0.079	−0.095	−0.211	−0.338	0.183	0.189	0.949
	coef.	0.088	−0.023	−0.102	−0.138	−0.117	−0.262	−0.378	0.116	0.126	0.864
12-05	z-value	40.0	−0.881	−4.34	−6.54	−14.1	−9.56	−20.5	7.67	7.17	21.0
	.025	0.079	−0.032	−0.162	−0.208	−0.149	−0.241	−0.357	0.164	0.142	0.704
	.975	0.088	0.012	−0.061	−0.112	−0.113	−0.159	−0.295	0.277	0.249	0.849
	coef.	0.084	−0.010	−0.112	−0.160	−0.131	−0.200	−0.326	0.221	0.196	0.776
12-12	z-value	50.4	2.24	−5.75	−5.67	−14.5	−9.99	−18.8	9.54	7.03	21.2
	.025	0.090	0.003	−0.193	−0.181	−0.147	−0.239	−0.309	0.205	0.131	0.673
	.975	0.098	0.044	−0.095	−0.088	−0.112	−0.161	−0.250	0.312	0.233	0.810
	coef.	0.094	0.023	−0.144	−0.135	−0.130	−0.200	−0.280	0.258	0.182	0.741
12-19	z-value	58.5	5.83	−4.41	−8.35	−11.6	−5.79	−16.7	9.75	9.96	21.1
	.025	0.092	0.038	−0.145	−0.225	−0.112	−0.145	−0.257	0.209	0.205	0.656
	.975	0.099	0.077	−0.056	−0.139	−0.080	−0.072	−0.203	0.315	0.305	0.791
	coef.	0.096	0.058	−0.101	−0.182	−0.096	−0.108	−0.230	0.262	0.255	0.723
12-26	z-value	59.7	7.60	0.214	−8.18	−10.5	−3.74	−13.5	8.16	12.2	17.7
	.025	0.096	0.061	−0.040	−0.221	−0.113	−0.114	−0.224	0.188	0.292	0.596
	.975	0.103	0.103	0.050	−0.136	−0.078	−0.036	−0.167	0.307	0.404	0.744
	coef.	0.099	0.082	0.005	−0.179	−0.096	−0.075	−0.196	0.247	0.348	0.670
01-02	z-value	83.3	12.3	−0.219	−10.6	−10.6	−1.50	−10.5	13.0	16.4	13.4
	.025	0.114	0.103	−0.047	−0.260	−0.108	−0.065	−0.168	0.324	0.398	0.421
	.975	0.119	0.142	0.037	−0.179	−0.074	0.009	−0.115	0.439	0.507	0.565
	coef.	0.117	0.122	−0.005	−0.219	−0.091	−0.028	−0.141	0.382	0.453	0.493
01-09	z-value	87.4	15.7	3.36	−11.1	−5.58	−1.64	−10.8	10.7	22.7	13.7
	.025	0.108	0.124	0.026	−0.236	−0.057	−0.061	−0.152	0.238	0.521	0.392
	.975	0.113	0.160	0.100	−0.165	−0.028	0.005	−0.106	0.344	0.619	0.523
	coef.	0.110	0.142	0.063	−0.200	−0.043	−0.028	−0.129	0.291	0.570	0.457
01-16	z-value	93.7	20.9	7.51	−11.2	2.99	−3.14	−12.9	10.8	26.0	12.2
	.025	0.108	0.164	0.099	−0.224	0.007	−0.083	−0.173	0.239	0.598	0.337
	.975	0.112	0.198	0.168	−0.157	0.036	−0.019	−0.127	0.345	0.695	0.466
	coef.	0.110	0.181	0.134	−0.191	0.021	−0.051	−0.150	0.292	0.647	0.401
01-23	z-value	82.7	20.1	12.9	−14.4	4.49	−0.739	−9.71	9.10	28.8	9.84
	.025	0.100	0.166	0.188	−0.270	0.019	−0.045	−0.137	0.206	0.706	0.274
	.975	0.105	0.202	0.255	−0.205	0.048	0.020	−0.091	0.320	0.808	0.410
	coef.	0.102	0.184	0.221	−0.238	0.034	−0.012	−0.114	0.263	0.757	0.342
01-30	z-value	81.5	17.6	19.7	−18.5	7.79	−2.38	−10.3	5.89	34.7	7.97
	.025	0.096	0.147	0.283	−0.317	0.043	−0.072	−0.140	0.117	0.866	0.211
	.975	0.101	0.184	0.346	−0.257	0.072	−0.007	−0.095	0.234	0.970	0.348
	coef.	0.099	0.166	0.315	−0.287	0.058	−0.039	−0.118	0.175	0.918	0.280

Coefficients with p-value in $[0.01, 0.05)$ are colored blue, and those with p-value ≥ 0.05, red. All other p-values are < 0.01.
